# Recent Insights into Eco-Friendly Extraction Techniques for Obtaining Bioactive Compounds from Fruit Seed Oils

**DOI:** 10.3390/foods14132271

**Published:** 2025-06-26

**Authors:** Sandra Rodríguez-Blázquez, Esther Gómez-Mejía, Noelia Rosales-Conrado, María Eugenia León-González

**Affiliations:** Department of Analytical Chemistry, Faculty of Chemistry Sciences, Complutense University of Madrid, 28040 Madrid, Spain; egomez03@ucm.es (E.G.-M.); nrosales@ucm.es (N.R.-C.)

**Keywords:** agri-food residues, fruit seed oils, bioactive compounds, green extraction techniques, design of experiments, green metrics, artificial intelligence, industrial applications

## Abstract

The valorization of agri-food waste has emerged as a global priority. In this context, fruit seed waste is being investigated for oil extraction due to its richness in bioactive compounds with remarkable health benefits. This review (2020–2025) focuses on the current state of eco-friendly extraction techniques for obtaining high-yield oils enriched with compounds such as tocopherols, polyphenols, fatty acids, phytosterols, and carotenoids. A comparison of the present method with conventional extraction techniques reveals several notable distinctions. Conventional methods are generally characterized by prolonged extraction times, elevated temperatures, and high amounts of solvents and/or energy. The findings of this review suggest that the extraction methodologies employed exerts a substantial influence on the yield and bioactive composition of the oil, which in turn affects its health-promoting properties. Furthermore, the results have demonstrated that alternative methodologies (microwave-assisted extraction, ultrasound-assisted extraction, pressurized liquid extraction, electric pulse extraction, enzyme-assisted extraction, subcritical extraction, and combinations thereof) have analogous oil yields in comparison with conventional methods. In addition, these oils present a superior bioactive profile with feasible potential in industrial and health applications. The novelty of this work lies in its emphasis on the valorization of fruit seed waste, as well as its sustainable approach. This sustainable approach utilizes experimental design strategies, the implementation of developments that employ comprehensive ecological metrics, and the latest trends in the application of artificial intelligence.

## 1. Introduction

Vegetable oils are natural extracts derived from seeds, fruit pulps, pits, sprouts, or tubers of various plants, which serve as nutritionally dense sources of energy [1]. Beyond their caloric value, these oils are rich in bioactive compounds that offer a wide range of health-promoting properties, including antioxidant, antibacterial, antifungal, anticancer, anti-inflammatory, and neuroprotective activities [2,3]. Furthermore, their versatility has led to the widespread use of vegetables oils across multiple industries, from nutrition and healthcare to cosmetics and everyday products such as varnishes, adhesives, synthetic resins, lubricants, soaps, paints, and bactericides [1,4].

Driven by global population growth, rising economic standards, and increasing awareness of the health benefits of vegetable oils, global demand continues to surge. In 2019–2020 alone, global consumption reached 200.27 million metric tons [1]. Meeting this growing demand, both for industrial applications and human consumption, requires the production of high-quality oils in substantial quantities. At the same time, consumers are increasingly seeking foods that not only satisfy sensory and nutritional expectations but also produced through environmentally responsible processes [4]. Preserving a greater proportion of bioactive compounds in the final product thus presents a significant challenge for the industry [1,4].

Among the various stages involved in vegetable oil processing, conventional techniques such as cold mechanical pressing are still widely used [1]. However, these methods often yield low oil recovery, as a significant portion remains trapped in the solid residue. To improve yields, organic solvents are commonly employed to extract the remaining oil. Yet, there is growing interest in reducing solvent use due to concerns over high removal costs, toxicity, safety risks, and environmental pollution [1]. In response, innovative extraction methods have been explored, particularly those utilizing waste materials from commonly consumed fruits such as avocados, cherries, plums, peaches, *citrus* fruits, and tropical varieties. These by-products represent an abundant and cost-effective source of bioactive-rich vegetable oils. Converting food waste into valuable bioproducts like these oils is a cornerstone of advancing global sustainability and fostering a circular economy. This waste management strategy also aligns with the United Nations’ Sustainable Development Goals (SDGs) outlined in the 2030 Agenda, promoting sustainable practices and reducing environmental impact [5,6].

This has spurred the development of innovative and environmentally friendly extraction technologies, including ultrasound-assisted extraction (UAE) [7], microwave-assisted extraction (MAE) [8], pressurized liquid extraction (PLE) [7], pulsed electric fields (PEFs) [9], subcritical extraction [10], and enzyme-assisted extraction (EAE) [11], as well as hybrid and sequential approaches, such as the combination of supercritical fluid extraction and ultrasound-assisted extraction (SFE-UAE) [12] or the complex pulsed electric field and supercritical fluid extraction (PEF-SFE) [9] or ultrasound-microwave assisted extraction (UMAE) [13]. These techniques aim to maximize the recovery of bioactive compounds while minimizing environmental impact, by shortening extraction times, reducing solvent use, and optimizing solvent nature, to name just a few.

Currently, many of these methods are being optimized or developed at the laboratory scale. As such, it is essential not only to assess their extraction efficiency but also to evaluate their overall sustainability. In this regard, chemometric tools such as experimental design, along with modeling and predictive tools based on artificial intelligence, can play a crucial role in determining the practical feasibility of large-scale implementation and identifying optimal operating conditions.

Accordingly, in light of the aforementioned considerations, the primary objective of this review is to provide a critical and comprehensive analysis of green and emerging technologies for the extraction of bioactive compounds from fruit seeds, an agri-food waste that holds significant untapped potential. The scope encompasses a detailed evaluation of current eco-friendly extraction strategies, with particular attention given to hybrid techniques, the integration of simulation tools for assessing the sustainability of developed processes, and the application of artificial intelligence as both a predictive and transformative tool to enhance process scalability and maximize the extraction efficiency. From this targeted perspective, this review aims to underscore both the technological advances and sustainability implications of these approaches, offering insights into their practical applicability and limitations.

**Figure 1:**
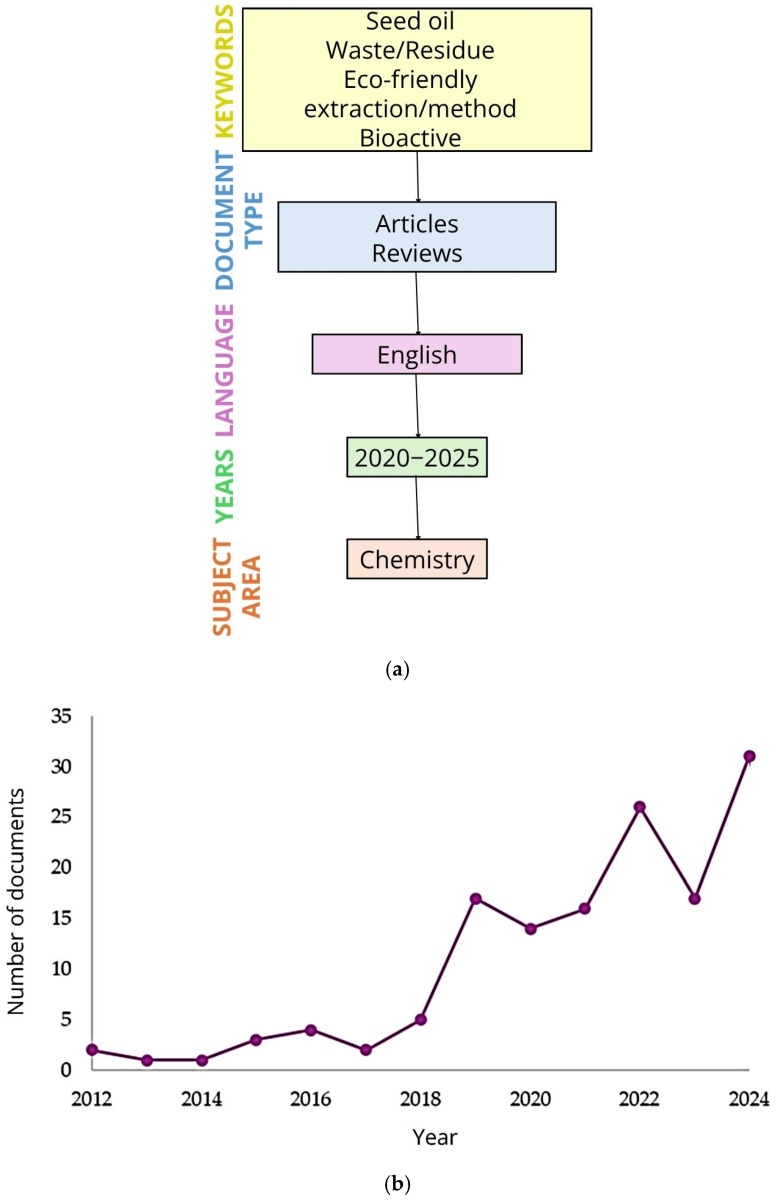
(**a**) Flow diagram illustrating the bibliographic selection process employed in this review. (**b**) Annual distribution of documents retrieved from Scopus using the keyword sequence outlined in the Methodology section.

The selection of studies was primarily conducted using the Scopus and ScienceDirect databases. The search strategy employed the following sequence of keywords: (seed oil), (waste or residue), (eco-friendly extraction or method), and (bioactive). Only articles and review papers published in English between 2020 and 2025 were considered. Additionally, the subject area was restricted to ‘Chemistry’, and duplicate entries were removed to avoid redundancy (Figure 1a). Studies offering substantial contributions to the field were selected for in-depth analysis, with a particular focus on those involving the extraction of oils from food waste sources such as plum seeds, citrus seeds, and avocado seeds, among others. As shown in Figure 1b, the annual distribution of publications retrieved from Scopus demonstrates a clear upward trajectory in research activity within this field since 2012. Although minor fluctuations are observed, the overall trend remains positive, culminating in 31 publications by 2024.

**Figure 2:**
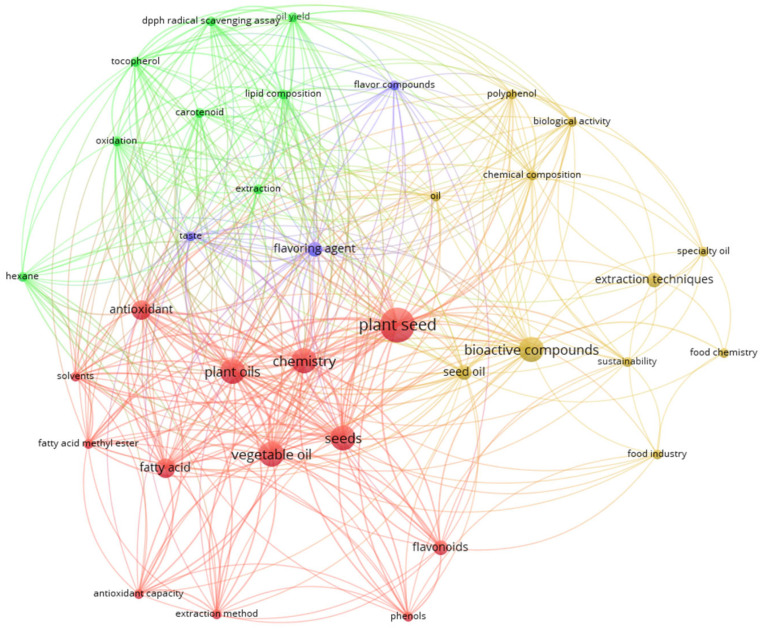
Keyword co-occurrence map generated using VOSviewer software (version 1.6.20), based on 150 documents retrieved from Scopus between 2012 and 2025. Clusters and their corresponding colors were automatically assigned according to a minimum threshold of three co-occurrences among the selected keywords (“seed oil”, “waste or residue”, “eco-friendly extraction or method”, and “bioactive”).

As a complementary element, Figure 2 displays a network of keywords identified based on co-occurrence within the analyzed literature, considering a minimum threshold of three occurrences for the selected terms (“seed oil”, “waste or residue”, “eco-friendly extraction or method”, and “bioactive”) between 2012 and 2025. The connecting lines illustrate the relationships among the keywords, while the size of each circle indicates the relative frequency or weight of the term. Greater distances between circles reflect a lower degree of correlation or thematic association. As can be seen, it reveals significant thematic intersections among terms such as “plant seed” (red cluster), “lipid composition” (green cluster), “flavour compounds” (blue cluster), and “food chemistry” (yellow cluster). Moreover, the term “extraction” appears in distinct configurations—specifically as “extraction techniques” and as “extraction”—within two separate clusters, represented in yellow and green, respectively. This distinction underscores the influence of the employed technique on both the chemical composition of the extracted compounds and the sustainability of the process.

On the other hand, Figure 2 highlights the conceptual interconnectivity and thematic clustering of these domains, reflecting an interdisciplinary approach to the study of bioactive compound and plant-derived oil extraction. Notably, these topics frequently co-occur with keywords such as “vegetable oil”, “plant seed”, “seed oil”, and “plant oils” within a more specific context denoting various bioactive compounds including fatty acids, phenols, flavonoids, tocopherols, and carotenoids (see red and green clusters). Furthermore, these terms are interconnected with themes related to extraction techniques, “chemistry”, “food industry/chemistry”, and “sustainability”, underscoring the pivotal role of analytical chemistry in the development and optimization of sustainable and innovative extraction methodologies that can significantly impact and contribute to the functioning of the agri-food industry.

## 2. Valorization of Fruit Seeds as Agri-Food Residues

The fruit and vegetable industry generates a substantial amount of waste, including peels, seeds, pomace, and pulp, which can account for between 25% and 30% of the total product volume. This waste is often classified as industrial waste, and its improper disposal can have a negative environmental impact. Furthermore, it should be noted that many of these products contain valuable bioactive substances. In the case of seeds, lipids, phytosterols, tocopherols, phenolic compounds, and carotenoids, among others, can be found [14]. In many cases these fruit seeds are discarded, and that is an economic loss and an environmental issue. The utilization of edible seeds for oil extraction has a long historical precedent, primarily due to their high lipid content. However, many seeds, including those from grapes and pomegranates, have not been employed in a commercial capacity for oil extraction until sufficient information regarding their potential health benefits has become available [15]. The extraction of bioactive compounds from agri-food waste has applications in various industrial sectors, including food, pharmaceuticals, and cosmetics. However, the sustainable extraction of these compounds presents various challenges. These include selective extraction to avoid the co-extraction of undesirable substances, the possibility of degradation of the compounds subject to extraction, or the consumption of solvents in some extraction procedures and the need for a balance between the costs and benefits of the overall extraction process [5]. To obtain extracts that can be used at an industrial level, conventional extractions include Soxhlet extraction, cold pressing, or supercritical CO_2_ extraction. These complete extracts have been used as such in the pharmaceutical industry in herbal preparations, as food supplements, or as ingredients in cosmetics. Not only do conventional techniques require a long extraction time and high energy consumption, but also a large volume of solvent in the case of Soxhlet extraction. To obtain higher bioactive recoveries, to use smaller amounts of solvents, or even to use so-called green solvents, unconventional extraction techniques have been employed both on a laboratory scale and on an industrial scale [16,17].

Each method of oil extraction has advantages and disadvantages from an environmental, practical, and economic point of view. Information on the environmental impact between extraction methods is limited, although there is scientific evidence on the adverse effects of solvents such as *n*-hexane. Another aspect that must be considered when implementing an extraction technique is economic viability, especially when it is desired to develop on an industrial scale. A techno-economic analysis on benefits and costs will include the valorization of by-products and the added value that can be derived from a higher quality of the oil by co-extraction of other compounds. It is imperative to consider the preservation of easily degradable compounds in certain experimental conditions that are indispensable to the extraction process. Such conditions may encompass elevated temperatures or pressure, among others [18]. For all these reasons, further research is required to study its practical and economic feasibility at an industrial level.

The efficacy of the extraction technique used in the isolation of compounds of interest from biological matrices is paramount. Consequently, a range of technologies have been developed for the extraction of bioactive compounds from diverse types of seeds that have not traditionally been utilized for oil extraction. A notable illustration of this phenomenon is the extraction of oil from the seeds of passion fruit by means of cold pressing [19,20], by industrial-scaled hydraulic pressing [21], by low-temperature continuous phase transition [22], by ultrasound-assisted extraction [11], by continuous expeller press [23], by supercritical CO_2_ extraction assisted by ultrasound [12], by subcritical fluid extraction [24], or by surfactant assisted aqueous extraction [25]. Other seeds widely studied for oil extraction and valorization are pomegranate [26,27] and citrus seeds [28,29,30], using various methods such as MAE, UAE, extraction in supercritical fluid or subcritical fluid and/or combined with EAE. As an example, Table 1 includes different pomegranate seed oil extraction procedures. It is evident that the extraction yield and the time required for extraction can vary significantly. This variation is attributed to the type of solvent or solvents employed, as well as the geographical origin of the sample.

Other oils have been extracted using supercritical fluids under different conditions from seeds such as *Sapucaia* (*Lecythis pisonis*) nuts [35], *Pracaxi* (*Pentaclethra macroloba*) seed [36], blackberry seeds, [32,37], *Butia odorata* seed [38], apple seeds [39], cherry seed [40] and raspberry seed [41].

**Figure 3:**
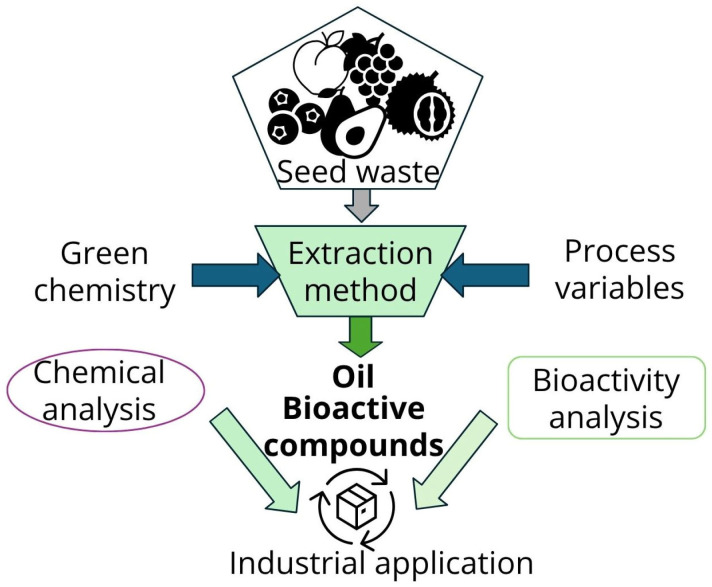
Waste seed recovery scheme.

In all cases, before choosing the extraction method, the complex nature of the seeds must be considered, since the selectivity of the extraction will be given both by the selected extraction method and its extraction conditions and by the nature of the matrix on which the extraction is applied. A considerable number of the methods employed are founded, at least in part, on the principles of green chemistry, which include the elimination of toxic solvents and minimal energy consumption. In numerous instances, the outcomes are contrasted with those attained by conventional extraction techniques. Nevertheless, the evaluation of the activity of the extracts and their chemical composition are the key to developing industrial applications that allow for the valorization of these wastes (Figure 3).

## 3. Bioactive Compounds in Fruit Seed Oils: Nutritional Value, Application Potential, and Determination Methods

**Figure 4:**
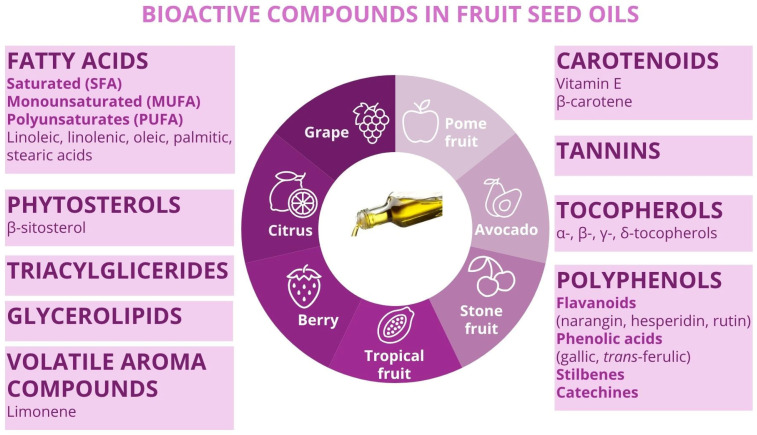
Representative bioactive compounds present in fruit seed oils.

Many fruit seed oils are nutritionally rich, containing high levels of bioactive compounds including antioxidants, vitamins, essential fatty acids, and phytonutrients, thereby rendering them suitable for diverse applications in food, nutraceuticals, cosmetics, and functional products (Figure 4) [14].

Several factors have been reported to influence the quality, yield, and potential applications of fruit seed oil. These include preharvest conditions—such as the cultivar and the fruit’s growing region—as well as the processing steps involved in oil production, including seed drying, pretreatment, and extraction methods. Together, these factors have a significant impact on the oil’s chemical composition and functional properties [42].

Grape seeds, a by-product of winemaking and juice production, are among the most valuable components derived from grapes. They contain approximately 7% to 20% of oil, depending on the variety and processing methods. Grape seed oil is notably rich in saturated (SFAs), monounsaturated (MUFAs), and polyunsaturated fatty acids (PUFAs), along with lipophilic compounds such as vitamin E and phytosterols. In addition, it contains a wide range of hydrophilic bioactives, including polyphenols (such as flavonoids, phenolic acids, stilbenes, catechins, and epicatechins), carotenoids, and tannins. These diverse constituents contribute to the oil’s potent antioxidant, anti-inflammatory, anticancer, and antibacterial properties, as well as its potential in preventing hypertension and supporting cardiovascular health. As a result, grape seed oil holds great promise for use in dietary supplements, anti-aging skincare products, therapeutic formulations, and other health-promoting applications [14]. Grapefruit seed oil is particularly notable for its high content of linoleic acid, β-sitosterol, and α-tocopherol, as well as flavonoids such as naringin, hesperidin, neohesperidin, and rutin. It also contains phenolic acids, with *trans*-ferulic acid and gallic acid being the most prominent. Additionally, a total of 26 volatile aromatic compounds have been identified in grapefruit seed oil, including d-limonene, furfural, and 3-methylbutanal [43].

*Citrus* fruit seeds are also valuable sources of edible oil, particularly rich in unsaturated fatty acids, including essential omega-6 and omega-3 fatty acids. In addition to these, *citrus* seed oils contain significant amounts of bioactive compounds such as tocopherols (vitamin E), limonoids, carotenoids, and phenolic compounds—primarily flavonoids. The high tocopherol content, combined with these phenolics, contributes to the oil’s strong antioxidant capacity, helping stabilize unsaturated fatty acids and providing effective free radical scavenging activity. As a result, *citrus* seed oils offer various health benefits, including cardiovascular disease prevention and immune modulation in inflammatory conditions. Notably, lemon seed oil contains a high concentration of limonene, a compound that has shown promise in early-stage cancer treatment [14], while sweet orange seed oil is a promising nutritional source with prominent antioxidant potential. It contains high levels of unsaturated fatty acids, particularly MUFA and PUFA types such as oleic, linoleic, and α-linolenic acids, all of which are associated with potential health benefits in humans [30]. Mandarin (*Citrus reticulata*) and lemon (*Citrus limon*) seed oils are characterized by the presence of key fatty acids, including linoleic, oleic, palmitic, α-linolenic, and stearic acids. Furthermore, the citrus oils also contain triacylglycerols, notably monolinolein and dilinolein derivatives [29].

Regarding tropical and exotic fruit seeds, they emerge as promising sources of functional and edible vegetable oils due to their high lipid content, offering valuable opportunities for selective use as industrial raw materials. The dominant fatty acids typically found in these oils are palmitic, oleic, and linoleic acids. Lipidomic studies have shown that passion fruit, cherimoya, and durian seeds are particularly rich in glycerolipids, sphingolipids, and fatty acyls, respectively, while litchi seeds contain high levels of glycerophospholipids and saccharolipids. Moreover, oils extracted from passion fruit and cherimoya seeds are especially rich in UFA, making them well suited for daily dietary lipid intake in alignment with internationally recognized dietary patterns such as the Mediterranean diet [44].Vitamin E and β-carotene have been also determined in passion fruit seed oil [22], as well as oleamides (particularly 9-octadecenamide) [32] and volatile aroma compounds [20]. Oil extracted from yellow passion fruit (*Passiflora edulis var. flavicarpa*) seeds is rich in unsaturated fatty acids and tocopherols. It also exhibits strong antioxidant properties and notable antibacterial activity against *Escherichia coli*, *Salmonella enteritidis*, *Staphylococcus aureus*, and *Bacillus cereus* [24].

Furthermore, dragon fruit seeds are highly nutritious, particularly in terms of essential fatty acids, and the use of seed oils has become increasingly popular. The oil primarily contains PUFAs, with linoleic acid being the most abundant. It also includes various tocopherol groups, such as α-tocopherol, γ-tocopherol, and δ-tocopherol. Dragon fruit seed oil is known for its anti-aging properties, helping to smooth and firm the skin. Rich in antioxidants, it is a valuable addition to creams, lotions, and moisturizers. In addition, due to its high levels of polyphenols and unsaturated fats, this oil holds significant value and can be microencapsulated for use in a variety of products [8]. Conversely, seed oil from cactus tropical fruit has also exhibited important amounts of linoleic acid, α-tocopherol, and canolol phenolic acid [45].

On the other hand, berry seeds are an important by-product, with their oils offering a rich source of essential nutrients. The primary fatty acids found in most berry seed oils are linoleic acid, linolenic acid, and oleic acid, resulting in low n−6/n−3 ratios. These oils typically contain around 90% UFAs, with a high concentration of PUFAs. Following oil extraction, significant amounts of antioxidants, phenolic compounds (such as phenolic acids and flavonoids), vitamins, phytosterols, and carotenoids remain in the oils. Berry seed oils are also rich in tocopherols, particularly α- and δ-tocopherol, with β-sitosterol being the primary sterol. Additionally, some berry seed oils are excellent sources of vitamins C and E, enhancing their value in the food, pharmaceutical, and cosmetic industries. These oils also exhibit antioxidant, antimicrobial, and anti-carcinogenic properties [14]. Particularly, blackberry seed oils have exhibited high contents of unsaturated fatty acid (mainly linoleic acid), β-carotene [37], polyphenols (mainly vanillin), and oleamides (9-octadecenamide) [32].

Stone fruits, belonging to the Rosaceae family, are distinguished by a hard pit or endocarp shell that surrounds the seeds or kernels. The most common stone fruits include almonds (*Prunus dulcis*), peaches (*Prunus persica*), nectarines (*Prunus persica* var. *nucipersica*), plums (*Prunus domestica*), cherries (Sweet: *Prunus avium* L; Sour: *Prunus cerasus* L.), and apricots (*Prunus armeniaca*). Seed shells contain high concentrations of toxic amygdalin, particularly in apricots. This toxicity limits the use of stone fruit seed oils in the food industry, driving interest in their potential applications in biodiesel production and cosmetics. The oils are rich in fatty acids and γ-tocopherol, along with sterols, primarily β-sitosterol, although some stone fruit seed oils are also distinguished for their high levels of potassium, magnesium, and B-complex vitamins [14]. In general, stone fruit oils and, particularly, plum seed oils, have exhibited good antioxidant activity and oxidative stability, thus showing potential application in the cosmetic and nutraceutical industries [2,46]. Additionally, cherry seed oil, which is rich in fatty acids, particularly linoleic, oleic, palmitic, and stearic acids, as well as γ-tocopherol, has demonstrated strong antioxidant potential, making it also suitable for use in the food and pharmaceutical industries [40].

In contrast, research on pome fruit seed oils has been limited, primarily due to the small size of the seeds. However, apple and pear seed oils have been found to be rich in fatty acids, particularly oleic and linoleic acids, as well as tocopherols (α-, β-, γ-, and δ-tocopherols) and carotenoids. These oils exhibit notable antibacterial and antioxidant properties, along with potential cytotoxic effects against certain cancer cells [14]. Specifically, apple seed oils are rich in antioxidant phenolic compounds such as phlorizin, phloretin, naringenin, vanillin, ferulic acid, and 4-hydroxybenzoic acid [39]. Pear seed oil is also viable and useful for sustainable biodiesel production [47], while pomegranate seed oil has been abundant in PUFAs (especially punicic acid), triacylglycerol molecules, squalene, β-sitosterol, and tocopherols (α, γ, and δ), thus exhibiting high nutritional properties [26].

Furthermore, the avocado processing industry generates a substantial amount of by-products, including avocado seeds. Avocados are well known for their rich nutritional profile, particularly due to their high content of MUFAs, especially oleic acid. The oil content of avocados is also of great significance, as they are rich in lipophilic components such as fatty acids (palmitic, oleic, and linoleic), tocopherols, and sterols. The hydrophilic components of avocados, including polyphenols and carotenoids, provide numerous health benefits and have proven effective as natural preservatives and antibacterial agents. Moreover, avocado oil has shown antidiabetic and hypotensive effects, mainly attributed to key compounds like stigmasterol and campestanol [48].

Otherwise, when determining the bioactive compounds in fruit seed oils, both the spectrophotometric and chromatographic methods are commonly used. Thus, the total polyphenol content (TPC) is often estimated using the simple and rapid Folin–Ciocalteu assay, by measuring the absorption of a blue complex at 765 nm, after an incubation period, and by employing gallic acid as standard [22,29,30,39]. However, this method does not differentiate between individual polyphenolic compounds and can be affected by other reducing substances present in the sample. In contrast, profiling the phenolic compounds involves identifying and quantifying individual polyphenols, typically achieved through high-performance liquid chromatography (HPLC) or ultra-HPLC coupled to diode-array (DAD) [39,43] or mass spectrometry (MS) [45] detectors. Separations are mainly conducted in the reversed phase under gradient elution mode, using non-polar C18 stationary phases and mixtures of organic solvents (acetonitrile or methanol) with additives such as ammonium acetate [45], formic acid [45] and sulphuric acid [43], or mixtures of methanol and water [39], as the mobile phase. While more specific and sensitive than spectrophotometric assays, HPLC methods require more advanced techniques and expertise.

α-, β, γ-, and δ-Tocopherol levels are usually determined using liquid chromatography in both the reversed phase [24,43,45] and normal phase modes [26,40], often with DAD [24,43] and fluorescence [8,26,40,45] detection. Common mobile phases are constituted with tetrahydrofurane–hexane [40], dioxane–hexane [8], and isopropanol–hexane [26] mixtures in the normal phase and methanol–water [43,45] and methanol–isopropanol [24] in the reversed phase mode.

Spectrophotometric methods are also used to assess the β-carotene content in fruit seed oils by measuring the absorbance of diluted samples in *n*-hexane [37,49] or dichloromethane [22] at 450 nm.

For fatty acid determination, gas chromatography (GC) is employed after a derivatization step to obtain fatty acid methyl esters (FAMEs), followed by temperature programming and detection through MS [22,28,32,50] or flame ionization detection (FID) [8,26,29,37,40,43,44,45]. Several reagents have been employed to obtain derivatized fatty acids, such as sodium hydroxide–methanol [44,50], boron–trifluoride methanol [40], BF_3_ methanolic [30,44], sodium methoxide [26], and KOH methanolic [28] solutions. A wide range of columns have been also used for the determination of FAMEs, including SH-Rtx-Wax (30 m × 0.32 mm ID × 0.25 µm) [37], SP-2560 (100 m × 0.25 mm ID, 0.20 µm) [40], RTX-wax (30 m × 0.25 mm ID × 0.25 μm) [30], CP-Sil 88 (60 m × 0.25 mm ID × 0.20 μm) [26] (100 m × 0.25 mm ID × 0.20 μm) [24], Agilent HP-50 (30 m × 0.32 mm ID × 0.25 μm) [8,45], cyanopropyl methylpolysiloxane HP-23 [29], HP-5MS UltraInert (30 m × 0.25 mm ID × 0.25 μm) [22], DB-5 ms (30 m × 0.25 mm × 0.25 μm) [32], HP-88 (100 m × 0.25 mm ID × 0.20 μm) [43], and SUPELCOWAX (30 m × 0.25 mm ID × 0.25 μm) [28] fused silica capillary columns.

## 4. Conventional Extraction Methods: Limitations and Challenges

The extraction of fruit seed oils is typically accomplished through conventional methodologies, encompassing mechanical pressing, solvent extraction, and supercritical CO_2_ extraction. It is evident that these methodologies have attained a high level of industry-wide adoption; nevertheless, it is imperative to acknowledge their inherent limitations [1,51]. Hence, this section discusses the principles of each extraction method, the factors influencing extraction yield, and the effect of the extraction method used on the bioactive composition of the oils, as well as the limitations and challenges of each extraction method.

### 4.1. Mechanical Pressing

Mechanical pressing is a solvent-free method that is often used for the extraction of oils from seed samples. The conventional mechanical extraction technique for oil production offers numerous benefits, including the superior quality of the extracted oil, the expeditious nature of the extraction process, the absence of organic solvents, which promotes process sustainability, and its straightforward scalability for industrial implementation. Nonetheless, the process is accompanied by several disadvantages such as low capacity per batch, low oil recovery percentage, high energy consumption, and high dependence on moisture content [1,51].

**Figure 5:**
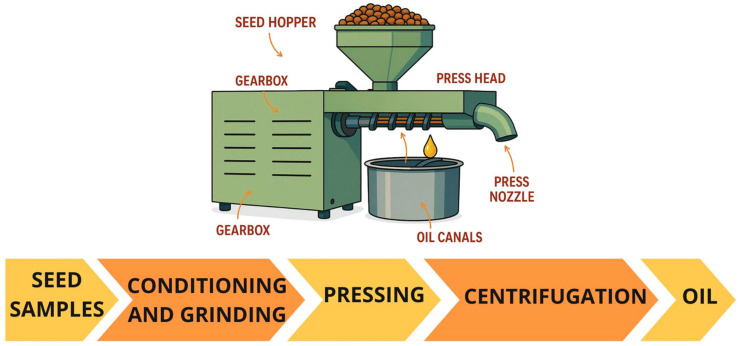
Flow chart of oil extraction through mechanical pressing.

The process relies on the mechanical pressing of seeds, divided into two main stages: material preparation and subsequent oil extraction (Figure 5). The preparation of the seed material consists of cleaning, preheating, breaking, and/or crushing [18,51]. Subsequently, as shown in Figure 5, seeds are collecting and pressing into the barrel surrounding the screw and the resulting oil is collected in a basin under the screw, and the defatted seeds is discharged at the end of the screw. Finally, due to the high content of impurities in the oil (such as pieces of non-extracted seeds) the oil is centrifuged and separated from the pellet [1].

According to mechanical pressing, different factors directly affect the oil recovery, such as the moisture content of the seed samples, the pretreatment of the seeds (e.g., roasting), the pressure, and the temperature. With regard to moisture content, normally an increase in moisture content has different effects on oil yield depending on the seed species studied. In general, an increase generally leads to a decrease in oil yield. Hasanov et al. [31] showed that by increasing flaxseed moisture content from 6 to 11–15.57% the oil recovery decreased moderately from 13.17 to 4.34%. They demonstrated this finding on the basis that higher moisture content in the raw material could exert a lubricating effect and significantly reduce the friction inside the press, thus resulting in lower oil yield. Another crucial factor when controlling the mechanical pressing is the pressure of the system. In general, the more pressure applied to the system, the higher the oil extraction yield [52]. Niu et al. [53] demonstrated that roasting tomato seeds previously at 130 to 170 °C significantly increased the oil content, increasing from 18.72 to 22.16%. This phenomenon was explained by the fact that the heat treatment process promoted oil release by decreasing enzymatic activity, coagulating proteins, and inducing structural changes in the seeds. The final factor to be considered in mechanical seed pressing is the selected extraction temperature. According to this factor, two distinct types of mechanical pressing are typically employed in the extraction of seed oils. These methods are referred to as cold pressing and hot pressing, respectively. The process of hot pressing involves the extraction of the desired substance at temperatures that typically range from 90 to 100 °C [54]. Cold-pressed oils are well known to be healthier because they have higher concentrations of phenolic compounds, tocopherols, phytosterols, fatty acids, and carotenoids, whereas bioactive compounds in hot-pressed oils are more prone to degradation due to the elevated temperatures used in this technique [55]. However, hot pressing correlates with higher oil extraction yields, with up to 80% of the oil present in the oilseed being recovered [54]. Specifically, Zhen et al. [56], found that the oil yield in flaxseed oil was significantly different using cold pressing (22.2%) and hot pressing, with the highest oil yield being obtained in hot pressing (32.7%).

Cold pressing has been widely employed for the extraction of fruit seed oils, such as pomegranate seeds, cherry seeds, passion fruit seeds, and lemon seeds, among others. The related information taken from the literature data is shown in Table 2.

### 4.2. Solvent Extraction

**Figure 6:**
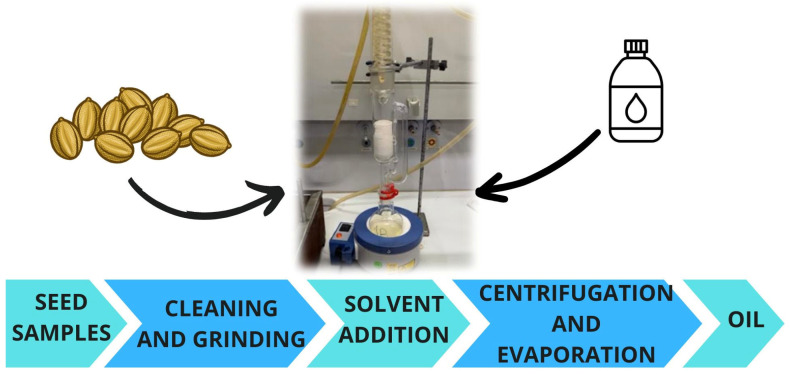
Flow chart of solvent oil extraction.

Solvent extraction is another traditional seed oil extraction. When comparing with the mechanical pressing, it is less sustainable, although this extraction technique allows for higher oil yields (oil recovery rate from 90 to 98%). However, this technique necessitates a substantial solvent demand and significant time and energy expenditure. The fundamental principle underlying this extraction technique involves the utilization of a solvent that comes into direct contact with the seed to extract the oil. In certain instances, the application of heat and agitation may be employed to enhance the extraction process. Once the solid–liquid extraction is completed, the mixture is centrifuged to remove the defatted seed from the oil phase, and then the organic solvent is evaporated from the oil (Figure 6) [1,62]. Based on this principle, there are several methodologies that can be employed (e.g., maceration, Soxhlet extraction, shaking extraction, and distillation) [1].

Oil recovery by solvent extraction mostly depends on the particle size of the oilseed, as well as the extraction temperature, the type and nature of the solvent (polar and non-polar), the solid–solvent ratio, and the extraction time [55].

Soxhlet extraction is the main classical methodology for obtaining vegetable oils from the oleaginous matter of seeds. This technique allows for the extraction of oils with high efficiency and reproducibility, especially in laboratory and research contexts, but its use is limited on an industrial scale, being replaced by continuous extraction methods that are more efficient and compatible with large-scale production. The solvent selection for this methodology is mainly based on the highest leaching properties of the required solute substrate. Among the main solvents used in seed oil extraction are *n*-hexane, ethanol, methanol, chloroform, diethyl ether, petroleum ether, and acetone. Gonfa Keneni et al. [63] studied different solvents (*n*-hexane, ethanol, diethyl ether, and heptane) for the Soxhlet extraction of oils from Jatropha seeds and observed that the highest extraction yield was obtained using *n*-hexane (41.24%). Liu et al. [26] evaluated the effect of *n*-hexane or petroleum ether on the recovery of pomegranate (*Punica granatum* L.) seed oil by Soxhlet extraction and observed that *n*-hexane was the most effective. Giuffrè et al. [64] used various solvents (acetone, ethyl acetate, chloroform, and petroleum ether) for tomato seed oil recovery by Soxhlet extraction and demonstrated that the highest oil yield was achieved using petroleum ether (21.2%).

Hexane is the most widely used solvent for oil extraction globally, due to its advantageous properties such as easy recovery of the extract, low latent heat of vaporization (330 kJ·kg^−1^), a narrow boiling point range (63–69 °C), excellent solubilizing capacity, high extraction efficiency, and its non-polar character. Notwithstanding, this solvent has many disadvantages such as its flammable properties with potential health hazards [18].

Owing to its high efficiency in lipid solubilization, *n*-hexane has been established by the Association of Official Analytical Collaboration (AOAC) as the standard solvent in Method 960.39 for the determination of oils and fats from seeds by conventional extraction [65]. In addition, Table 3 summarizes some data from the literature using this methodology to obtain fruit seed oils.

### 4.3. Supercritical CO_2_ Extraction

Supercritical CO_2_ extraction (ScCO_2_) is widely used today for extracting oils from seeds and is considered a sustainable method. This method uses variations in temperature and pressure to bring a gas to its supercritical state—a phase in which the properties of gases and liquids merge, making the two phases indistinguishable [1].

According to Versteeg et al. [69], ScCO_2_ is a separation technique based on mass transfer, where convection within the supercritical solvent phase (CO_2_) typically serves as the dominant mechanism for transporting solutes. This methodology has several strengths in comparison with solvent extraction. The absence of residual solvents, the low temperature applied during the separation of the seed materials, the enhancement of diffusivity, which allows for greater selectivity, and the reduction in extraction times are all factors that must be considered [70]. In addition, Morya et al. [55] exposed that supercritical CO_2_ extraction enables obtaining an oil quality similar to that of the mechanical pressing process explained above.

**Figure 7:**
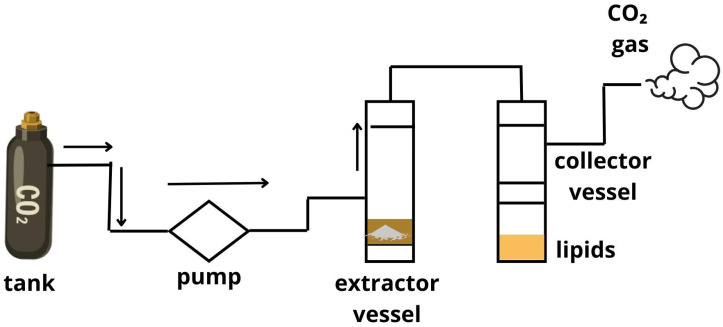
Flow chart of supercritical CO_2_ extraction.

The typical system comprises a CO_2_ tank, a pump, an oven containing a sample vessel, an extractor, and a collector. The liquid CO_2_ is pumped to the extractor containing the seed, where the oil is dissolved. The resulting mixture then passes into a low-pressure chamber where the oil is separated from the CO_2_. The CO_2_ is converted into a gas, which can then be recycled or released [69,70]. The flow chart of the system is represented in Figure 7.

The efficiency of the oil extraction process using this system depends on the temperature, the pressure, and the solubility of the oil in the extraction fluid, as well as the contact time between the oil material and the extraction fluid. Particularly, Pavlić et al. [41] used a Box–Behnken design to optimize the raspberry oil extraction method using kinetic models of equal extraction and showed that the highest extraction efficiency with the maximum initial rate of matter transfer was achieved at higher levels of pressure (35 MPa) and CO_2_ flow rate (0.4 kg CO_2_·h^−1^). Concurrently, it was ascertained that temperature (40 °C) and particle size (200–400 μm) levels required maintenance at low levels.

In addition, a quite common practice to improve extraction efficiency is the use of co-solvents such as ethanol or *n*-hexane. The observed effect can be attributed to the enhanced solubility of lipids. This increase in solubility may result from a higher density in the ScCO_2_ and co-solvent mixture, or from specific intermolecular interactions between the co-solvent and certain solutes. Using co-solvent can be beneficial in separation processes, especially when it improves selectivity by interacting specifically with target compounds. Even so, the overall solubility of all components in the mixture also tends to increase due to the rise in fluid density [70]. Specially, Souza Correa et al. [71], demonstrated that the use of ethanol as a co-solvent during supercritical CO_2_ oil extraction with berry seeds provided a higher oil yield, as well as greater efficiency in the extraction of bioactive components (e.g., fatty acids and phenolic compounds) as well as enhanced antioxidant activity. In other study, Macawile et al. [72] proved that the efficiency of oil extraction from *Gliricidia sepium* seeds using supercritical CO_2_ extraction with *n*-hexane as a co-solvent significantly improved due to the increased solubility of lipid components.

Furthermore, recent studies to optimize the supercritical extraction process tend to resort to artificial intelligence simulators. These include artificial neural networks (ANNs). Specifically, Dimić et al. [34] carried out a study to develop and optimize the supercritical CO_2_ extraction of cherry seed oil using sequential extraction kinetics modeling and an ANN. The resulting Box–Behnken design demonstrated that the highest oil recovery was obtained at 350 bar, 50 °C, and 0.4 kg CO_2_. In addition, an ANN was applied to calculate the initial mass transfer rate, as it allowed for the simulation of the initial phase and to control the extraction process.

Nowadays, this methodology has emerged as a traditional method for extracting oils from fruit seeds. Therefore, Table 4 shows a summary of the literature data that apply this methodology in the extraction of fruit seed oils as well as the composition of the bioactive compounds present in them.

However, despite the advantages of this technique such as the selectivity and sustainability of the process, this technique requires a high application cost of ScCO_2_ which restricts its use mainly to oils produced in small quantities [69]. Consequently, the development of new sustainable alternatives for oil extraction is required.

## 5. Recent Advances in Eco-Friendly Extraction Methods and Factors Affecting Extraction Efficiency and Oil Quality

**Figure 8:**
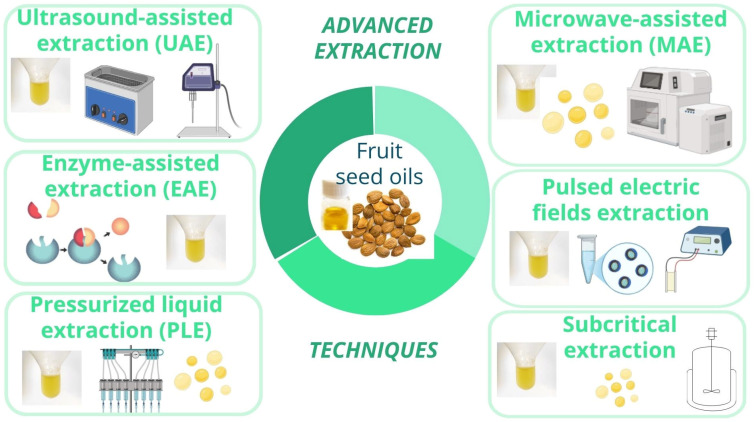
Advanced sustainable extraction methods for fruit seed oil recovery.

As previously reported, traditional extraction methods often involve the use of harmful solvents, long processing times, and high temperatures, which can degrade heat-sensitive compounds. To address these challenges, advanced extraction techniques such as UAE, MAE, EAE, PLE, and subcritical extraction, as well as the novel PEF technique have been employed to efficiently recover bioactive compounds from fruit seeds (Figure 8).

In many cases, it is necessary to use hybrid or combined extraction techniques, which involve the coupling of two or even three methods, to enhance extraction efficiency.

### 5.1. Ultrasound-Assisted Extraction (UAE)

The utilization of ultrasound-assisted extraction in the extraction of bioactive compounds is consistent with several principles inherent to green chemistry. These principles include the enhancement of operator safety, the reduction in energy consumption, the effective management of waste, and the minimization of the use of toxic or environmentally harmful solvents [76].

UAE is distinguished by the implementation of high-frequency waves, which are utilized to induce the formation of cavitation bubbles within the solvent. The subsequent collapse of these bubbles results in the generation of high temperatures and localized pressures, which in turn modify the sample matrix and enhance the extraction efficiency. Once the extraction is completed, the oil mixture requires a centrifugation or filtration step in order to eliminate the defatted seeds and then the solvent is evaporated using a rotary evaporator or under vacuum at a low temperature.

The main advantages of UAE are focused on reducing solvent consumption, the time needed to extract, and improving extraction efficiency [77]. The extraction conditions using UAE depend on the bioactive compounds to be extracted and the material from which they must be extracted. A number of authors have evaluated experimental designs and response surface analyses with the objective of obtaining optimal extraction conditions. They have also sought to derive equations that would allow for the relating of the experimental parameters used with oil extraction performance or other factors, such as oxidative stability [11,12]. The breakdown of cell structures and cell walls in plant samples produces an improvement in the extraction of bioactive compounds so that the efficiency of the extraction depends on the power of the ultrasound, the frequency, the duty cycle, the extraction time, and the solvent selected, so the optimal extraction conditions must be evaluated for each type of bioactive compound and each type of sample. Deep eutectic solvent extraction and ultrasound-assisted extraction have been performed in order to improve the oil and amygdalin extraction performance in bitter apricot kernels. The combined effect of UAE and the mixture of choline chloride–lactic acid facilitate the breakage of the apricot kernels, which would explain the increase in extraction yield [78]. In other study, Marinaccio L. et al. [79] used a natural volatile deep eutectic solvent (NADES), specifically menthol–thymol 1:1, for ultrasound-assisted grape seed oil extraction and compared it with UAE using *n*-hexane as a solvent. Their findings demonstrated that the phenolic content increased significantly (18.65 mg GAE·g^−1^), as well as the α-linolenic acid content (5.18%). Meanwhile, the oleic and linoleic acid content decreased in the oil extracted using NADES.

In the optimization process, it is important to consider that increasing the power of the ultrasound can enhance the extraction of some compounds, but it may also have a negative impact on others. While an increase in acoustic power and extraction time can facilitate mass transfer, it can also potentially lead to an increase in the formation of free radicals [45] These free radicals, in turn, have the potential to degrade certain bioactive compounds, such as polyphenols, or to induce the extraction of unwanted compounds, like amygdalin [39]. It is important to note that the UAE also depends on the frequency used. Flexible plant materials are best suited to a frequency range of 20 to 40 kHz. However, for more rigid materials, a frequency range of up to 500 kHz may be necessary [77]. The extraction of fatty acids in oil is strongly influenced by their affinity for the solvent, but it is also affected by the time of exposure to ultrasound and by the content of SFAs and UFAs in the sample [39].

The ultrasound waves can be generated in a water bath or by using a probe immersed in the sample. While the ultrasonic bath can be used for multiple samples, the probe sonicator can only be applied to one sample at a time. It should also be considered that the intensity provided by the bath ultrasound [30] is lower than that provided for the probe [12,27,80].

Compared to oil extraction by conventional methods such as stem distillation or mechanical pressing, ultrasonically assisted extraction allows for lower energy consumption and a reduction in solvent or substitution with green solvents and an extraction yield of the same order of magnitude. Table 5 shows the conditions used to extract oil with bioactive compounds from various seed residues. In a more advanced situation, ultrasound can be combined with other technologies like microwave [13], enzymatic [27], and supercritical fluid extractions [12] to be used directly in the process or could be used as a pretreatment process. These hybrid techniques are useful in increasing the extraction efficiency for oil [26,27,81].

### 5.2. Microwave-Assisted Extraction (MAE)

Microwave-assisted extraction has emerged as a compelling and increasingly adopted non-conventional technology for oil extraction. It operates through the application of non-ionizing electromagnetic radiation (ranging from 300 MHz to 300 GHz) directed at polarizable materials, including both the solvent and the oil-bearing matrix. This radiation induces internal heating via ionic conduction and dipolar rotation, leading to the rupture of plant cell walls as a result of vapor pressure buildup. Such structural disruption facilitates the rapid release and dissolution of oil into the solvent, thereby enhancing extraction efficiency [14,82]. The rapid heating characteristic of MAE enables the production of high-quality oils in significantly reduced timeframes and with minimal solvent usage, an advantage that positions it favorably against conventional solvent-based techniques such as Soxhlet extraction [1,3,14].

Unlike traditional methods, MAE does not necessitate prior thermal treatment of the raw material, offering both economic and energy-saving benefits for industrial applications. Although microwave-assisted extraction has demonstrated considerable potential in the valorization of agro-industrial seed residues—such as grape seeds [83,84], pomegranate seeds [26], and dragon fruit seeds—its application remains relatively scarce. This underutilization persists despite promising results in terms of oil yield and bioactive compound recovery, as summarized in Table 6, which outlines the extraction conditions and key outcomes for various seed types.

Be that as it may, microwave energy can serve as a pretreatment step in other extraction processes [1,14]. For instance, the use of microwave drying and heating as a prior step for oil recovery from avocado seeds was reported by Satriana et al. [85], and Bose et al. [33] reported a twofold increase in oil yield from mechanically pressed tropical seeds (*Simarouba glauca*) following microwave pretreatment. Nevertheless, as previously noted, the use of microwaves solely as a pretreatment is generally less efficient, both economically and energetically, than their direct application as extraction technique.

**Table 6:** MAE conditions for the extraction of agri-food seed oils.

Food Waste	Solvent	Processing conditions	Oil Recovery (wt%)	Bioactive Compounds	References
Pomegranate seeds	*n*-hexane or petroleum ether	Power: 500 W Frequency: 40 kHz Time: 9 min; Sample-to-solvent ratio: 1:6 (*w*/*v*)	11.72–12.47	Palmitic acid: 29.3% Oleic acid: 49.5% Stearic acid: 19.9% Linoleic acid: 53.6% Punicic acid: 776.5% α-Eleoestearic acid: 5.3% β-Eleoestearic acid: 33.0% Catalpic acid: 10.2% Arachidic acid: 6.2% Arachidonic acid: 7.6% ꞵ-Sitosterol: 3.47 mg·g^−1^ Squalene: 1.59 mg·g^−1^ α-tocopherol: 17.24 mg·100 g^−1^ γ-tocopherol: 322.7 mg·100 g^−1^ δ-tocopherol: 8.23 mg·100 g^−1^	[38]
	*n*-hexane	Power: 220 W, Time: 5 min; Sample-to-solvent ratio: 1:10 (*w*/*v*)	35.19	Palmitic acid: 2.04% Oleic acid: 4.10% Stearic acid: 1.71% Linoleic acid: 3.84% α-Linolenic acid: 86.53% Margaric acid: 0.04% Arachidic acid: 0.37% Gadoleic acid: 0.69.0% TPC: 7.42 mg·g^−1^	[86]
Dragon fruit seeds	-	Power: 450 W; Time: 10 min	34.3	Palmitic acid: 14.69% Stearic acid: 7.30% Oleic acid: 18.66% Linolenic acid: 52.14% Arachidonic acid:0.04% TPC: 96.71 mg GAE·g^−1^ α-tocopherol: 52.19 mg·kg^−1^ γ-tocopherol: 921.58 mg·kg^−1^ δ-tocopherol: 76.6 mg·kg^−1^	[8]

In summary, MAE offers numerous advantages, including shorter processing times, lower operational costs, compact equipment requirements, reduced solvent consumption, and higher yields. However, despite its many benefits, MAE may not be universally suitable for all plant matrices. Excessive microwave energy can compromise plant tissue integrity, and the elevated temperatures reached during the process may degrade thermolabile bioactive compounds, thereby diminishing oil quality and bioactivity. Consequently, MAE is not recommended for the extraction of oils rich in PUFAs, which are particularly prone to oxidative degradation [5,14].

### 5.3. Enzyme-Assisted Extraction (EAE)

Enzyme-assisted enzyme extraction relies on the enzymatic pretreatment of seeds to release substances that bind to cell walls in order to increase the extraction of compounds of interest. The extraction of oil from seeds is a challenging process due to the complexity of the cell wall composition, which consists of cellulose, hemicellulose, lignin, and pectin. The application of specific enzymes, such as carbohydrates, that are capable of degrading the cell membrane, has been shown to facilitate the release of oil. Proteases have been demonstrated to play a pivotal role in the hydrolysis of cell membrane proteins, thereby enabling the efficient extraction of oil. From an ecological perspective, it is imperative to acknowledge that enzymatic treatment is conducted at relatively low temperatures, thereby eliminating the generation of solvent residues. A potential disadvantage of enzyme use is the cost, which can be significant. However, this can be mitigated by increasing the extraction yield or by reusing immobilized enzymes. In the extraction process, it is crucial to consider the working pH of the enzyme and the isoelectric point of the proteins (pI) that constitute the seed, since, on the one hand, if the pH is not adequate, the enzyme can be deactivated, and, on the other hand, if the pH is close to pI, precipitation of the seed proteins can occur [14,18].

**Figure 9:**
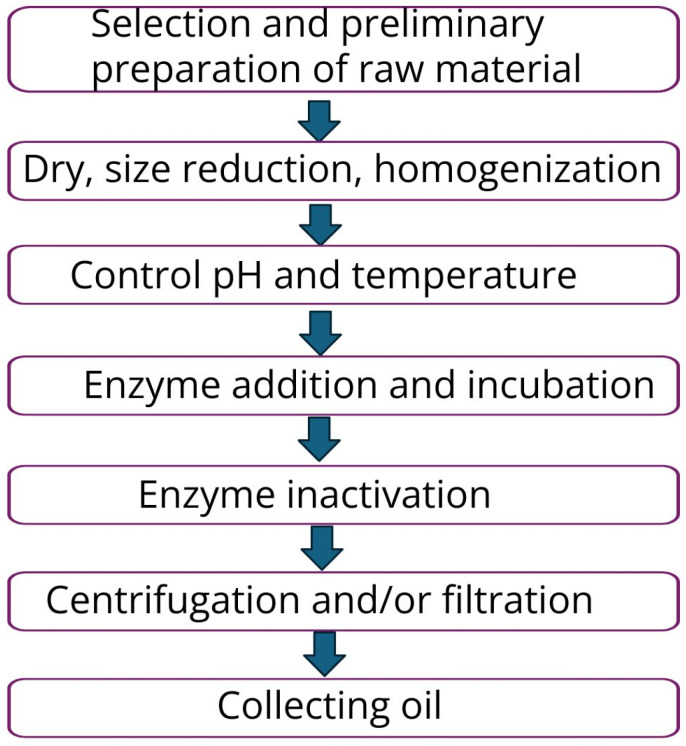
Enzyme-assisted extraction main steps.

In most cases, enzyme-assisted extraction consists of the stages described in Figure 9 [87]. Some of these steps are common with other extraction methods, but in this case pH and temperature control are essential parameters in process optimization.

The use of proteases for the joint extraction of oils and proteins in seeds/grains of fruits such as mango, lemon, or pumpkin has been evaluated to improve the performance of protein and fat extraction. The oil extracts showed a high nutritional value as they had a high content of unsaturated fatty acids. In the procedure, an enzyme–substrate ratio of 1:100 (*w*/*w*) was used in a phosphate buffer at a pH of 7.5 and hydrolyzed at 60 °C for 16 h. To deactivate the enzyme, it was heated for 10 min at 90 °C and finally centrifuged to separate the different phases [28]. With a focus on increasing the extraction yield and decreasing the time of extraction, enzyme-assisted extraction has been combined with ultrasound-assisted extraction using enzyme treatment as a pre-step or using ultrasound stirring during the incubation process [27,77].

### 5.4. Pressurized Liquid Extraction (PLE)

PLE is a technique that uses a solvent in its liquid state under elevated temperatures (typically between 27 and 227 °C) and pressures (from 5 to 20 MPa) but below the solvent’s critical point. These subcritical conditions enhance solvent penetration into the solid plant tissue and disrupt the binding interactions between the target compounds and the matrix, thereby accelerating the extraction process.

Considered a sustainable method, PLE minimizes solvent use, reduces energy consumption, and is easily automated, all of which contribute to its low environmental impact. Ethanol and hydroalcoholic mixtures are commonly used as solvents because they are considered green solvents and are easy to remove. Their adjustable polarity makes them versatile solvent systems for various extraction purposes.

In addition, PLE can be conducted in either intermittent or dynamic modes. In intermittent extraction, the system is pressurized for a single cycle, which is often repeated several times to improve extraction efficiency. This approach offers significant benefits, such as shorter extraction times and reduced solvent consumption, leading to energy and solvent savings. In contrast, dynamic extraction involves the continuous flow of solvent through the raw material bed, enabling the constant recovery of extracts and the collection of different fractions during the process. However, continuous solvent flow can lead to unnecessary dilution of the extract if the extraction time is extended. Increasing the solvent flow rate can reduce extraction time, which is particularly advantageous for thermo-sensitive compounds, as it minimizes their exposure to heat.

On the other hand, key process variables—such as temperature, pressure, solvent flow rate, extraction time, solvent choice, solvent-to-feed mass ratio (S/F), moisture content, and particle size—are crucial to the efficiency of the technique. Thermal effects impact the process by reducing solvent viscosity, weakening matrix–compound interactions, and altering solvent properties. Moreover, continuous solvent flow plays a vital role in maintaining constant interaction between the solvent and the processed material, which facilitates better mass transfer and extraction kinetics. Increasing solvent flow rates can significantly enhance the extraction yield. In terms of the extraction solvent, higher water content in hydroalcoholic mixtures improves the recovery of hydrophilic compounds but may reduce the extractability of non-polar lipids. It is important to note that higher moisture content leads to greater interaction with water, improving extraction efficiency when using water or hydroalcoholic mixtures as solvents. The S/F ratio also plays a critical role in balancing extraction efficiency with economic feasibility. Regarding particle size, reducing it increases the surface area available for extraction, enhances solvent penetration, and improves mass transfer. However, smaller particle sizes may lead to compaction, which can reduce solvent flow and decrease extraction efficiency.

The findings demonstrate that PLE is highly effective in extracting a wide range of bioactive compounds, including polyphenols, unsaturated fatty acids, tocopherols, and carotenoids. The resulting extracts typically exhibit significant anticancer, antidiabetic, antioxidant, and antimicrobial activities [48,88].

Compared to conventional methods, PLE offers several advantages, including shorter extraction times, reduced solvent consumption, high extraction efficiency, moderate environmental impact, and the possibility of solvent recycling. However, there are some drawbacks. These include potential analyte degradation under high pressure and temperature, the lack of miniaturized commercial devices that hinder its integration with other techniques, and the strong dependence of extraction efficiency on factors such as matrix type and the optimization of parameters like time and temperature. Additionally, the high cost of equipment and the removal of solvents remains a significant challenge for industrial-scale implementation [89,90].

However, PLE has been applied to recover valuable polyphenolic compounds from avocado seeds at temperatures up to 200 °C and pressures ranging from 11 to 22.5 MPa, using ethanol or hydroalcoholic mixtures. The major polyphenolic compounds recovered include procyanidins, and flavonoids (such as epicatechin, catechin, quercetin, and rutin) and their derivatives, as well as phenolic acids like chlorogenic, vanillic, and gallic acids [48].

Furthermore, Figueroa et al. [7] have employed an ethanol–water 50:50 (*v*/*v*) mixture at 200 °C and 11 MPa to efficiently extract organic acids, phenolic acids, flavonoids, catechins, and condensed tannins from avocado seeds. This method enabled obtaining extracts rich in 4-hydroxybenzoic acid with high antioxidant properties [7].

PLE has also been utilized to extract oils from the seeds of various berry fruits, including cranberries, black currants, red currants, strawberries, and chokeberries. The seed samples were mixed with diatomaceous earth, and the extraction was carried out in four 5 min cycles, using *n*-hexane as the solvent, at 150 °C and a pressure of 10.34 MPa. The primary fatty acids identified in the extracted oils were linoleic, α-linolenic, and oleic acids, which also exhibited high oxidative stability. Furthermore, the oils displayed low atherogenicity and thrombogenicity indexes, and, combined with high hypocholesterolemic index values, they are considered oils of high nutritional quality [91].

In order to summarize the PLE methods for the extraction of fruit seed oils, a summary of the literature data is shown in Table 7.

### 5.5. Pulsed Electric Fields (PEF) Extraction

Pulsed electric field technology has emerged as a cutting-edge, non-thermal, environmentally sustainable, and energy-efficient method for the extraction of bioactive compounds from biological matrices, making it particularly suitable for the recovery of oils from residual seeds. This technique is grounded in the principle of electroporation, wherein a high-intensity electric field is applied between two electrodes immersed in a solution containing the target material. The application of short-duration electric pulses, ranging from microseconds to milliseconds, induces electropermeabilization of cellular membranes, thereby facilitating the release of valuable intracellular compounds without the need for elevated temperatures. This characteristic is especially advantageous for preserving thermolabile substances. Unlike conventional thermal methods, PEF treatment maintains the organoleptic and nutritional qualities of food products, including the color, aroma, flavor, texture, and nutritional value, rendering it an appealing option for oil recovery processes intended for the food industry [4,5].

PEF systems typically comprise a high-voltage pulse generator, a monitoring and control unit, and a treatment chamber, the geometry and electrode configuration of which vary according to the processing mode. The effectiveness of PEF treatment is governed by several critical parameters, including the electric field strength (determined by the ratio of applied voltage to the distance between electrodes), the shape, number, and duration of pulses, the pulse frequency, and the specific energy, defined as the energy delivered per unit mass of the treated material. Collectively, these parameters not only enhance the efficiency and speed of the extraction process but also minimize waste generation and enable rapid processing. These attributes underscore the potential of PEF as a pivotal tool in advancing sustainable, high-quality processing technologies within the modern food industry [4].

Moreover, the non-destructive nature of PEF technology, coupled with its low energy demands, significantly enhances its suitability for industrial-scale applications [5]. However, its broader adoption continues to be limited by the substantial initial capital investment required for implementation. In this context, PEF has predominantly been employed as a pretreatment strategy for seeds, aiming to disrupt the cellular structure and thereby facilitate more efficient oil extraction in subsequent stages [92]. These subsequent extraction methods may include conventional techniques such as Soxhlet extraction and mechanical pressing, as well as alternative approaches like UAE [4]. For instance, [93] applied PEF to recover oils from berry seeds, including blackcurrant, redcurrant, raspberry, and chokeberry, using 2 g of sample material, electrode voltages of 8 or 10 kV, a pulse width of 7 µs, a frequency of 20 Hz, and a specific energy input of 50 kV·kg^−1^ over a 30 min treatment period. Among the tested samples, PEF proved most effective for enhancing oil yield from raspberry seeds, while no significant improvement was observed in the case of blackcurrant seeds. Notably, PEF treatment was found to improve the oxidative stability of the extracted oils without adversely affecting their thermal properties or fatty acid profiles, underscoring its potential as a gentle yet effective technique for high-quality oil recovery [93].

### 5.6. Subcritical Extraction

Subcritical fluid extraction is predicated on the utilization of solvents at temperatures between the boiling point and the critical temperature, and at a pressure below the critical point, with the capacity to maintain the solvent in a liquid state. Extractions with compressed solvents under subcritical conditions are considered green because the solvent can be reused and recovered. In the various subcritical fluids that have been utilized in the literature, *n*-butane and propane appear to be the most commonly employed in oil extraction processes. This preference is likely attributed to their colorless nature and cost-effectiveness. In addition to their excellent solubility of lipophilic compounds, they have the potential to be used in shorter extraction times and at lower critical pressures and temperatures, which may result in a higher-quality product [10].

Subcritical extraction is a simple process which has many astounding advantages in comparison with the traditional extraction techniques, including low pressure and temperature, a reduction in the extraction time, improved selectivity, and environmental compatibility, as well as eliminating toxic solvents. The parameters that govern the extraction process, including the extraction temperature, time, solvent ratio, particle size, and co-solvents, have been demonstrated to modulate the efficiency of subcritical extraction at low temperatures [94].

This emerging extraction technique has been used as a method for extracting fruit seed oil. In this particular case, Liu et al. [26] employed subcritical extraction with *n*-butanol to extract oil from pomegranate seeds. The experimental conditions encompassed a solid–solvent ratio of 1:6, wt.%, a temperature of 45 °C, an extraction time of 40 min, and a pressure of 0.6 MPa. A comparative analysis was conducted between this methodology and Soxhlet extraction as well as the sustainable MAE and UAE techniques. The findings indicated that this methodology resulted in the greatest increase in oil percentage (14.50%) compared to UAE and MAE. In addition, the oil extraction percentage obtained in this study was comparable to that achieved through Soxhlet extraction (15.66%) with *n*-hexane as a solvent. Furthermore, the oils exhibited comparable oleic (52.4 mg·g^−1^) and linoleic acid (53.7 mg·g^−1^) content to that obtained through Soxhlet extraction with *n*-hexane (55.4 and 57.5 mg·g^−1^, respectively). Furthermore, these samples exhibited higher concentrations of punicic acid (772.3 vs. 762.1 mg·g^−1^). Conversely, this method yielded higher levels of squalene, β-sitosterol, and α-, β-, γ-, and δ-tocopherols compared to Soxhlet extraction.

In addition, the use of water as a solvent for the subcritical extraction of essential oils from seeds has also been reported in the literature. In this case, this solvent allows for the extraction of higher amounts of polyphenols, terpenes, and other polar components [95].

In short, this emerging technique is currently being investigated for application with seed oils as an alternative to conventional methods.

### 5.7. Hybrid Extraction Technologies

The use of two or three techniques for the extraction of seed oils has been applied with two fundamental objectives: either to increase the efficiency of the extraction and/or to improve the extraction of bioactive products, which may be affected by the pressure conditions and/or temperatures used in various extraction techniques and which may lead to losses of antioxidant capacity or stability of the oil, among others [96].

Usually, to improve the extraction efficiency of bioactive compounds from seeds containing low levels of oil, a combination of conventional and alternative processes is required. In the case of passion fruit seed oil extraction, combining supercritical fluid extraction (at 40 °C and 16 MPa) with ultrasonic agitation (160 W) increased the overall yield to 20.9%, compared to 12.3% for SFE (at 40 °C and 16 MPa) without ultrasound-assisted extraction. This fact can be explained on the basis of the possible cavitation produced by the ultrasonic waves in the extraction bed, which favored the rupture of the sample matrix and the more favorable release of the oily phase. This combination of techniques must be previously optimized to find the conditions that produce a greater release of the extractable material from the seeds without increasing by too much the energy cost involved in the application of ultrasound. Although the oil yield increased, a decrease in γ-tocopherol (7.3 vs. 10.6 mg·100 g^−1^), γ-tocotrienol (27 vs. 36 mg·100 g^−1^), and δ-tocotrienol (34 vs. 45 mg·100 g^−1^) content was observed under these conditions. Meanwhile, an increase in oleic acid (19 vs. 15.9%) content and a decrease in linoleic acid (65 vs. 67.9%) was observed when ultrasound was applied in combination with SFE. This could be explained by the breakdown of the linoleic double bond when using a previous cavitation process by ultrasound [12].

In other study, Ćurko et al. [9] optimized a supercritical CO_2_ oil extraction method in combination with an electric pulse pretreatment from grape seeds. A Box–Behnken design was applied by studying the pressure, temperature, and flow rate factors of the SFE. The PEF pretreatments were performed at 5 kV·cm^−1^ and 120 hz for 5 and 1 min of extraction. The findings revealed that the PEF pretreatment in supercritical oil extraction significantly increased the oil yield to a value of 81.8 kg·g^−1^. Other authors in the literature concluded that the utilization of PEF as a pretreatment before the extraction process leads to the electrical decomposition of the cells and therefore to their increased permeability, thus favoring the extraction of the oil from the seed samples [92]. Furthermore, this research demonstrated that this hybrid technique significantly increased the content of sterols, tocopherols, polyunsaturated fatty acids, and phenolic acids compared to the untreated procedure.

As previously reported, the mechanical pressing technique is characterized by low extraction yields of the oils obtained. Therefore, the use of enzymes as a pretreatment phase could be a sustainable alternative, as they tend to soften and/or destroy the cellular structures, thus aiding in oil extraction [97]. In one research study, Candan et al. [98] developed a hybrid methodology with the combination of an enzymatic pretreatment prior to oil extraction by mechanical pressing in the recovery of grape, apricot, and flaxseed oils. Their results showed that enzymatic pretreatment and cold-press extraction showed positive results in terms of total carotenoids, tocopherols, oil yield, phenolics, antioxidant activity, and oxidative stability.

In other work, Bakhshabadi et al. [92] employed two pretreatment methodologies for black cumin seeds before cold pressing was employed, PEF (electric field strength of 3.25 kV·cm^−1^ and number of pulses of 30) and MAE (540 W for 180 s), to study the oil extraction efficiency. The achieved findings demonstrated that both of the pretreatments increased the oil extraction efficiency and its oxidative stability.

In addition to the combination of conventional and alternative methods. Recently, oil extraction processes have been developed by combining alternative sustainable methods (e.g., UMAE; aqueous enzymatic pretreatment microwave-assisted extraction (HCUEPM)) with each other.

In this line, ultrasound stirring in combination with microwave-assisted extraction (UMAE) has also been used to improve the extraction of kernel power oil. Specifically, Mwaurah et al. [13] noted that this combination resulted in an increase in extraction yield of 8.13%. This increase can be explained by the extraction method requiring two stages, the first involving the penetration of the solvent into the cell matrix and the second involving the diffusion of the extractable compounds through the porous cell wall to the solvent used for extraction. The first stage seems to be favored by ultrasound agitation. Furthermore, this hybrid technique allowed for the maintenance of the bioactive composition of the oils compared to the MAE methodology in the absence of UAE [13].

Alternatively, with a focus on enhancing oil extraction from *Litsea cubeba* seeds, Yang et al. [50] used homogenization circulating ultrasound in combination with an aqueous enzymatic pretreatment microwave-assisted extraction (HCUEPM). The enzymes, hemicellulose, pectinase, and neutral protease, yielded oils with a similar fatty acid composition, but the use of enzymes resulted in a higher yield in oil extraction using shorter extraction times.

In other research study, Hu et al. [99] optimized a simultaneous ultrasonic–microwave-assisted aqueous enzymatic extraction (UMAEE) for the extraction of cherry seed oils. Hence, a Plackett–Burman design and Box–Behnken design were employed, and the factors considered in this study were the ultrasonic power, the microwave power, the extraction time, and the enzyme concentration. Under the optimal extraction conditions (560 W, 323 W, 38 min, and 2.7%, respectively), the findings achieved an oil recovery rate of 83.85%. In addition, the fatty acid composition of the oil was not significantly modified in this new methodology in comparison to solvent extraction. However, it was characterized by better physicochemical properties and a higher content of bioactive compounds. It should be noted that the UMAEE technique is an efficient and environmentally friendly method.

Concluding all the results discussed above, Table 8 shows a summary of the bibliographic data applying hybrid techniques in the extraction of fruit seed oils as well as the composition of the bioactive compounds present in them.

## 6. Green Assessment of Extraction Techniques

**Figure 10:**
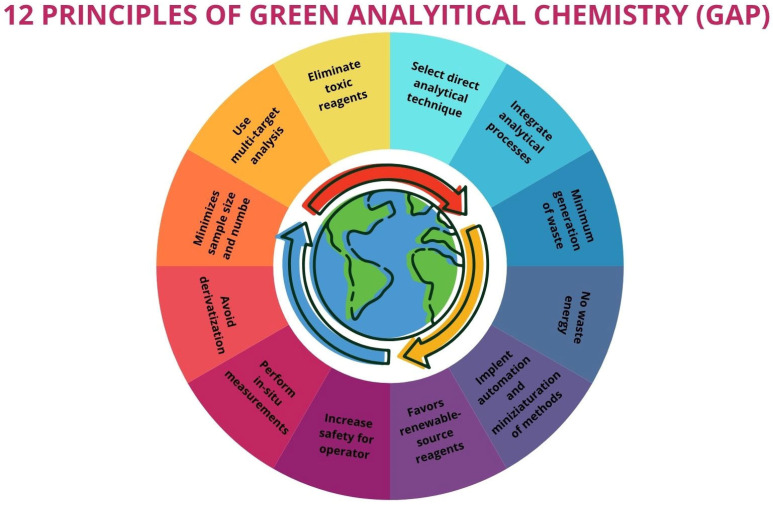
The 12 principles of Green Analytical Chemistry (GAC).

In recent years, given the increasing importance of sustainability in chemical processes, the design and application of Green Analytical Metrics (GAMs) has gained significant relevance. These metrics are essential tools for evaluating the environmental impact of analytical methods in alignment with the principles of Green Analytical Chemistry (GAC), as illustrated in Figure 10 [100].

Along these lines, to estimate the environmental impacts of chemical processes, several metrics are currently used that are distinguished by a different degree of sophistication, known as GAMs. These metrics consider relevant aspects such as waste generation, energy consumption, reagent toxicity, process efficiency, and operator safety [101]. These sustainability metrics in analytical chemistry have undergone significant advances since the introduction of the preliminary National Index of Environmental Methods (NEMI) in 2002. In recent years, several updated tools have been proposed in the literature, reflecting a broader and more refined approach to assessing ecology. These include the Eco-scale, the Green Motion tool, the E-factor, the Green Certificate, the Green Analytical Procedure Index (GAPI), its complementary version ComplexGAPI, the Analytical Greenness Metric Approach (AGREE), the Blue Applicability Grade Index (BAGI), and the Analytical Greenness Metric for Sample Preparation (AGREEprep) [102].

Among all these metric methodologies, the most widely used due to its inclusiveness in covering all GAC principles, as well as offering a comprehensive, flexible, and simple evaluation approach that provides easy-to-interpret and informative results, is AGREEprep. This methodology is widely used as a guide for the development of new methodologies that are more responsible and aligned with current sustainability objectives [103]. AGREEprep was created by Wojciech Wojnowski et al. [103] in 2022. This metric evaluates the sustainability of sample preparation methods in analytical chemistry using a 10-principle approach based on the 12 principles of green chemistry. Each principle is rated from 0 to 1, with 1 indicating the highest level of sustainability. Finally, an overall sustainability index (from 0 to 1) is generated that summarizes the overall performance of the method and is represented in a visual chart. In the charts generated by AGREEprep, the green areas represent high sustainability across the evaluated criteria, while the red zones highlight critical aspects that need improvement from an environmental perspective [101].

Specifically, Ferrara et al. [104] used the AGREEprep metric to compare different extraction and derivatization workflows for FAMEs in different classes of food samples. They demonstrated that this methodology enables the rapid and simple development of new approaches aligned with sustainability. They highlighted the factors of safety, ease of use, effectiveness, and compatibility of various sample matrices during sustainable oil extraction.

In other study, Rabiej-Kozioł et al. [105], two conventional methods of oil extraction from *Nigella sativa* L. seeds, namely cold pressing and solvent extraction, were evaluated in terms of their ecological factor using the AGREEprep method. The green scores calculated for oil extraction by cold pressing and extraction with *n*-hexane as a solvent were 0.79 and 0.25, respectively. This demonstrated the effectiveness of cold pressing as a sustainable method of oil extraction.

In a different research study, Costa et al. [106] performed the AGREEprep methodology to evaluate the methods applied in the preparation of vegetable oil samples: microwave-assisted extraction based on emulsion breaking (MAEB); emulsion breaking-induced extraction; microemulsion; and ultrasound-assisted extraction. Their results noted that both MAEB and microemulsion contributed significantly to the sustainability of the oil extraction process.

In this review, to assess the environmental performance of the advanced sustainable techniques reviewed, as compared to conventional extraction methods, a theoretical evaluation using AGREEprep was carried out based on the methodological information available in the literature. The results of this evaluation are presented in Table 9, which summarizes the performance of various oil extraction methodologies according to the ten AGREEprep criteria selected for analysis.

To emphasize the sustainability aspect, a weighting system was applied to each criterion, assigning the highest weight (score of 5) to criterion 2, which addresses the use of hazardous materials, followed by a score of 4 for both criterion 4, related to the non-generation of waste during the process, and criterion 8, concerning energy consumption.

A score of 3 was assigned to criterion 3 on sustainability, renewability, and reusability of materials, as well as to criterion 5 on sample size economy, criterion 6 on sample throughput, criterion 7 on method integration and automation, and criterion 10 on operator safety.

The lowest scores were given to criterion 1, which relates to the location of the sample analysis, and criterion 9, which evaluates the configuration of the sample for post-preparation analysis, as these were considered to have less influence on the overall environmental impact of the methodology.

As shown in the results presented in Table 9, all the advanced sustainable techniques examined in this review demonstrated a notably higher degree of sustainability, with AGREEprep scores ranging from 0.62 to 0.75, compared to conventional extraction methodologies, which exhibited lower scores between 0.20 and 0.64.

Although AGREEprep can significantly aid in evaluating the potential for industrial scalability of various methodologies, it should be viewed as a preliminary tool rather than a definitive solution. Its application offers valuable insight into how certain processes might perform at a larger scale and under industrial conditions. However, given that many of these methodologies are relatively recent and still under development, it is essential to continue conducting in-depth research [103].

## 7. Integrating Artificial Intelligence into Waste Valorization Processes

Food waste is a serious global issue, with significant economic, social, and environmental repercussions. The extensive waste generated across the entire food supply chain—from production to consumption—highlights the urgent demand for innovative and efficient management strategies. In this line, artificial intelligence (AI) has emerged as a powerful ally, thanks to its ability to process and interpret large volumes of data, recognize patterns, and forecast outcomes. Technologies such as machine learning and advanced data analytics can be leveraged to examine detailed information on waste production, composition, and valorization pathways. Specifically, there is a growing need to apply AI-driven tools in the treatment of seed-derived food waste to optimize oil extraction processes, enhancing resource recovery and promoting circular economy practices [109].

Machine learning represents a methodological shift towards systems that learn from data. In traditional programming, data and rules are provided to the computer, and then the expected response is calculated using the rules provided. However, in machine learning, both the data and the expected responses are provided to the model, and the rules are the result. These learned rules are then applied to predict the response for new data, thus enabling faster machine learning. There are two different ways in which the machine can learn, unsupervised and supervised learnings. On the one hand, supervised learning is a widely used machine learning approach in which the algorithm is trained with labelled data, e.g., data that includes both the input features and the correct output. The goal is to learn the correlations between the inputs and the corresponding outputs. This method is commonly used in tasks such as classification and regression. On the other hand, unsupervised learning is used when the samples do not contain labels. In this type of learning, the machine is allowed to discover hidden patterns in the data on its own, without the help of labels. The most common categories are clustering and dimension reduction [110]. Specifically, Iweka et al. [111] employed machine learning to optimize and predict the extraction of sustainable green oil from mature palm kernel seeds using the Soxhlet technique and observed that the value predicted by the model for the maximum yield was 38.0354% (wt) at 40 min of extraction time, 200 mL of solvent volume, and 60 g of sample weight.

Another type of artificial intelligence widely applied in the literature is artificial neural networks (ANNs), inspired by the structure and functioning of the human brain. In particular, an ANN also provides a robust approach to predicting oil extraction performance using various extraction techniques. By mimicking the way the human brain processes information, ANNs can analyze complex, non-linear relationships between input and output variables. This makes them particularly valuable for modeling and comparing different extraction methods, thereby assessing the sustainability of the process. ANNs can learn from experimental data to accurately predict performance and optimize process conditions with a focus on improving the efficiency of oil recovery [112]. Agu et al. [113] employed the response surface methodology (RSM) and an ANN to optimize the oil yield of *Terminalia catappa* L. seeds through Soxhlet extraction with n-hexane as the solvent. Under the optimum conditions for temperature, particle size, and extraction time, the RSM predicted an oil yield of 62.92%, which was validated as 60.34%, whereas the ANN-predicted yield was 60.39%, which was validated as 60.40%. The obtained optimization results noted that the ANN was a more effective approach than the RSM.

The current state of these artificial intelligence prediction methodologies is very new, so further study is required. The implementation of AI in the valorization of seed waste to obtain bioactive oils can be a powerful tool for predicting the oil recovery that would be obtained with different extraction methods, as well as predicting the bioactive compounds that would be obtained. However, its widespread adoption is currently limited. For large-scale operations, this can be a barrier due to the high cost of implementation. Furthermore, the complexity of integrating AI systems into existing agri-food infrastructure can also pose a significant challenge. In addition, ensuring data privacy and security is crucial for the ethical and responsible implementation of AI in the agri-food sector. Hence, further research and development are essential to overcome these limitations and enable broader adoption of AI in seed waste valorization and other sustainable practices. Collaborative efforts between industry and policymakers will be key to establishing standardized protocols, improving data accessibility, and reducing implementation costs. By addressing these challenges, AI has the potential to significantly enhance the efficiency and environmental performance of bioactive oil production, ultimately contributing to a more circular and sustainable agri-food system [109].

## 8. Industrial Applications and Future Perspectives

Although numerous authors have proposed potential applications for oils extracted via sustainable methods—emphasizing their bioactive properties, such as antioxidant and antibacterial activities, as well as their capacity to avoid the extraction of toxic or anti-nutritional compounds [20,21,35]—there is a limited number of studies that have developed these applications at an industrial or semi-industrial scale. Additionally, economic evaluations regarding the feasibility of their industrial-scale implementation are still limited [17].

Among studies evaluating the economic impact, the production of biodiesel from oil-rich waste, such as pear seeds via transesterification processes, stands out [47]. Other authors, using the circular economy criteria, have also obtained biodiesel by converting *Citrus aurantium* seed oil into methyl esters using recyclable zirconium oxide nanoparticles [114].

It is also worth noting the applications in the development of fungal control materials that extend the shelf life of foods such as strawberries, proposed by Silva et al. [19], using passion fruit seed oil together with other materials. Another novel application that may have an industrial application is to use oil from avocado seeds in a cold thermal storage system for food preservation [115].

In addition, the use of seed oils in the formulation of sunscreens is also noteworthy. Along these lines, Chu et al. [116] reported that pumpkin seed oil, due to its high content of fatty acids and squalene, could be used in the formulation of sunscreens. Moreover, Tsiapali et al. [117] pointed out that grape seed oil, due to its high content of omega-6 fatty acids, polyphenols, flavonoids, and tannins, is suitable for use in the formulation of sunscreens thanks to its added ability to delay skin ageing.

Another possible application for seed oils is their potential use as a functional food component. Afzal et al. [118] noted that sweet cherry oil obtained from food industry waste is a rich source of bioactive compounds, carotenoids, unsaturated fatty acids, and antioxidants. It is widely used as a preservative and functional food ingredient.

As for the future perspective for the valorization of oils produced from seed residues, work is underway to design new metrics that will facilitate informed decision-making for various industries, enabling them to prioritize more sustainable extraction practices. In this context, Path2Green was created by de Souza Mesquita et al. [119] to provide a holistic assessment of the sustainability of an extraction method, considering factors such as resource depletion, energy consumption, waste generation, and biodiversity preservation. However, it is necessary to further develop and refine these metrics to align with future needs and evolving regulatory frameworks.

Therefore, there is no doubt that the study of the properties of seed residue oils in the food industry can favor their potential application in industry, but there are still studies to be carried out on a semi-industrial scale to favor the integration of these oils in different industries. All of these alternative extraction technologies are currently known to be mature, offering significant intellectual property opportunities for specific applications. However, they are underutilized due to a lack of information on return on investment. In this line, initiatives are currently underway to conduct a technical–economic analysis (TEA) in order to predict the economic impact of each extraction technique [47]. Additionally, among the modeling options, Consequential Life Cycle Assessments (CLCAs) and Attributional Life Cycle Assessments (ALCAs) are of particular interest. These assessments predict the economic and environmental impact of each extraction process, thereby assisting in the design of appropriate strategic decisions.

Other relevant difficulties and barriers include a lack of technical experts and start-ups in the industry, poor competition among equipment suppliers, and a shortage of appropriate pilot facilities [17]. Furthermore, the entry of these technologies into the market is delayed due to the exhaustive safety, harmlessness, and quality testing required by the EFSA (European Food Safety Authority) or the FDA (Food and Drug Administration). Another major barrier to its industrial implementation is consumer acceptance. Although there is currently a growing trend toward natural products, the cost of accessing sustainable products is holding this trend back [120].

Notwithstanding, existing regulations increasingly emphasize the recognition and promotion of waste recycling and the enhancement of food system sustainability, aiming to facilitate its practical application across industries. Specifically, European Union policies support the development of new carbon-neutral labeling schemes, thereby strengthening the credibility and impact of sustainability initiatives (European commission 2024, [121]). Consequently, should these trends persist, it is anticipated that detailed information regarding each process for extracting oil from seed residues will become available in the future, thereby facilitating the implementation of these methodologies on an industrial scale.

## 9. Conclusions

This review underscores a prevailing tendency among researchers to devise more sustainable extraction methodologies and systematically evaluate the extracts obtained, with a view to correlating the bioactivity of the oils with the nature of the compounds extracted. A considerable proportion of the experimental developments incorporate optimization processes or the utilization of neural networks, which facilitate the establishment of experimental conditions conducive to achieving elevated oil extraction yields. Furthermore, these processes encompass the consideration of additional properties, including stability against oxidation and the presence of compounds exhibiting cardioprotective and antioxidant activity. These characteristics render these residues more appealing to industry. In the field of environmental science, there have been concerted efforts to develop processes that are more environmentally friendly. These efforts have involved the use of various programs that facilitate the scaling up of extraction procedures. The programs take into account multiple parameters related to environmental impact, thereby ensuring that the development of more environmentally friendly processes is informed by a comprehensive understanding of ecological considerations. Despite the existence of certain economic methodologies that facilitate the evaluation of the prospective industrial application, the majority of research endeavors concentrate on the optimization of extraction yield, the analysis of bioactive substances, and the comparison of these with conventional extraction techniques, eschewing economic comparisons between the disparate extraction techniques. In the preceding twelve-month period, the incorporation of artificial intelligence into customary extraction methodologies has been observed. Notwithstanding, it is anticipated that in the imminent future, the economic accessibility of generative artificial intelligence will be enhanced, rendering it a viable instrument for the assessment and refinement of more sustainable extraction techniques.

## Figures and Tables

**Table 1:** Effect of the extraction method on the oil yield of pomegranate seeds.

Extraction Method	Solvent	Extraction Time	Oil Recovery (wt%)	References
Soxhlet	*n*-hexane	8 h	15.66	[25]
Soxhlet	Petroleum ether	8 h	14.92	[25]
Shaking extraction	*n*-hexane	2 h	12.10	[25]
Shaking extraction	Petroleum ether	2 h	11.32	[25]
Mechanical pressing	-	8 min	9.00	[31]
MAE	*n*-hexane	9 min	12.47	[25]
MAE	Petroleum ether	9 min	11.72	[25]
MAE	*n*-hexane or petroleum ether	9 min	11.72–12.47	[32]
MAE	*n*-hexane	5 min	35.19	[33]
UAE	*n*-hexane	18 min	13.63	[25]
UAE	Petroleum ether	18 min	12.82	[25]
UAE	water	2–8 h	4.29–25.11	[26]
SubE	*n*-butane	40 min	14.5	[25]
Sc-CO_2_	CO_2_	2 h	16	[34]

**Table 2:** Literature data on the application of hot and cold pressing in the extraction of fruit seed oils.

Food Waste Material	Seed Mass (g)	Mechanical Pressing Method	Temperature Pressing (°C)	Oil Recovery (wt%)	Content of Principal Bioactive Compounds	References
Pomegranate seeds (*Punica granatum* L.)	200	Cold pressing YODA YD-ZY-02A (Yoda, Warsaw, Poland)	28–32	9	Linolenic acid: 80% Oleic acid: 5% Linoleic acid: 5%	[57]
Cherry seeds	6000	Cold pressing (SZR “Mikron”, Temerin, Serbia)	70	4	Linoleic acid: 46.82% Oleic acid: 41.92% α-tocopherol: 6.39 mg·100 g^−1^ ꞵ-tocopherol: 1.07 mg·100 g^−1^ γ-tocopherol: 25.22 mg·100 g^−1^ δ-tocopherol: 5.41 mg·100 g^−1^	[40]
Passion fruit seeds	200	Cold pressing (grain type MKS-J03)	62	6–12	Linoleic acid: 38–70% Oleic acid: 9–17%	[58]
Muskmelon (*Cucumis bisexualis*) seeds	100	Hot pressing (DH-50 screw oil press)	90	23	Oleic acid: 49.60% Linoleic acid: 26.60%	[59]
Lemon (*Citrus* limon L.) seeds	3000	Cold pressing (Koçmaksan, ESM 3710, İzmir, Turkey)	40	17	Palmitic acid: 20.88% Oleic acid: 30.86% Linoleic acid: 30.77% Beta-sitosterol: 76.55% α-tocopherol: 155 mg·kg^−1^	[60]
Berry seeds	n.d.	Cold pressing (IBG Momforts Oekotec, DD85G, Mönchengladbach, Germany)	50	n.d.	TPC: 8.9–19.3 mg GAE·100 g^−1^ Brassicasterol: <0.1–465.7 mg·kg^−1^ Campesterol: <0.1–200 mg·kg^−1^ Stigmasterol: <0.1–34 mg·kg^−1^	[61]

n.d.: not declared.

**Table 3:** Summary of literature data using Soxhlet extraction with *n*-hexane as a solvent for obtaining fruit seed oils.

Food Waste Material	Mass Seed to Solvent Ratio (*w*/*v*)	Time of Extraction (h)	Oil Recovery (wt%)	Content of Principal Bioactive Compounds	References
Pomegranate seeds (*Punica granatum* L.)	1:6	8	15.66	Punicic acid: 762.2 mg·g^−1^ Oleic acid: 55.4 mg·g^−1^ Linoleic acid: 57.5 mg·g^−1^ Squalene: 1.20 mg·g^−1^ ꞵ-Sitosterol: 3.23 mg·g^−1^ α-tocopherol: 17.66 mg·100 g^−1^ γ-tocopherol: 325.3 mg·100 g^−1^ δ-tocopherol: 8.33 mg·100 g^−1^	[26]
Passion seeds	1:20	6	26.12	Palmitic acid: 10.22% Oleic acid: 17.11% Linoleic acid: 68.97% α-tocopherol: 1.68 mg·100 g^−1^ γ-tocopherol: 1.62 mg·100 g^−1^ δ-tocopherol: 4.93 mg·100 g^−1^	[24]
*Citrus* seeds	1:20	20	24.94	Palmitic acid: 21.72% Oleic acid: 26.00% Linoleic acid: 34.32% ꞵ-Sitosterol: 32.25% Limonene: 5.1% ∆-Avenasterol: 5.69% Campesterol: 7.55% 1,2-Benzenedicarboxylic acid, mono(2-ethylhexyl) ester: 18.66%	[66]
Avocado seeds (*Persea Americana* L.)	1:10	8	41.3	TPC: 25.78 mg GAE·g^−1^ TFC: 72.69 mg QE·100 mg^−1^ Myristic acid: 33.33% Linoleic acid: 20.12% Oleic acid: 11.60% Palmitic acid: 7.07%	[67]
*Prunus* seeds (peach, apricot, plum, and cherry)	1:10	6	Peach: 30% Apricot: 38% Plum: 37.4% Cherry: 36.0%	Palmitic acid: 5.71–8.1% Oleic acid: 48.6–72.7% Linoleic acid: 16.4–39.3%	[46]
Apple seeds	1:15	10	22.5	Quercetin: 0.01 μg·g^−1^ *p*-coumaric: 0.098 μg·g^−1^ *Trans*-ferulic acid: 0.104 μg·g^−1^ Phloridzin dihydrate: 0.01 μg·g^−1^ Naringenin: 0.31 μg·g^−1^ Palmitic acid: 6.24% Stearic acid: 2.97% Oleic acid: 24.15% Linoleic acid: 49.03% Linolenic acid: 1.87% Arachidic acid: 1.22%	[68]
Cherry seeds	1:4	6	3.19	Linoleic acid: 41.44% Oleic acid: 47.17% α-tocopherol: 7.13 mg·100 g^−1^ ꞵ-tocopherol: 1.112 mg·100 g^−1^ γ-tocopherol: 31.47 mg·100 g^−1^ δ-tocopherol: 6.64 mg·100 g^−1^	[40]

**Table 4:** Literature review of the application of supercritical CO_2_ extraction in seed oil recovery.

Food Waste Material	Pressure (bar)	Temperature (°C)	CO_2_ Flow Rate (kg·h^−1^)	Particle Size (μm)	Oil Recovery (wt%)	Content of Principal Bioactive Compounds	References
Cherry seeds	200–350 bar	40–70	0.2–0.4	800	3.62–13.02	Oleic acid: 40.89–41.65% Linoleic acid: 46.32–47.37% α-tocopherol: 1.75–10.54 mg·100 g^−1^ ꞵ-tocopherol: 0.78–1.11 mg·100 g^−1^ γ-tocopherol: 6.23–40.60 mg·100 g^−1^ δ-tocopherol: 2.08–8.42 mg·100 g^−1^	[40]
*Citrus* seeds	200–250	45–60	1.62	n.d.	15.2–19.6	Palmitic acid: 17.68–20.01% Oleic acid: 29.51–30.67% Linoleic acid: 36–39.44% Limonene: 5.73–6.39% ꞵ-sitosterol: 26.63–28.37% 1,2-Benzenedicarboxylic acid, mono(2-ethylhexyl) ester: 26.55–30.34%	[66]
Berry seeds	1000	100	240	n.d.	12–18	α-tocopherol: 21.7–176.1 mg·kg^−1^ ꞵ + γ-tocopherol: 50.6–1027.7 mg·kg^−1^ δ-tocopherol: 2.1–1104.8 mg·kg^−1^ α-linolenic acid: 3.2–38.0% Linoleic acid: 47.9–72.9% Palmitic acid: 2.1–4.3% Oleic acid: 10.5–18.0%	[73]
Apple seeds	75–350	25–90	0.12–7.6	n.d.	20.5	Quercetin: 0.03 μg·g^−1^ *p*-coumaric: 0.17 μg·g^−1^ Trans-ferulic acid: 0.12 μg·g^−1^ Phloridzin dihydrate: 2.97 μg·g^−1^ Naringenin: 0.63 μg·g^−1^ Palimitic acid: 13.39% Stearic acid: 7.69% Oleic acid: 34.84% Linoleic acid: 63.76% Linolenic acid: 1.61% Arachidic acid: 2.04%	[68]
Pomegranate seeds (*Punica granatum* L.)	200	40	0.108	n.d.	16	Punicic acid: 80.7% Oleic acid: 1.58% Linoleic acid: 3.43% 2,4-Dihydroxyphenylacetic acid: 0.14 mg·100 g^−1^ Ferulic acid: 0.29 mg·100 g^−1^ *Trans*-Cinnamic acid: 0.14 mg·100 g^−1^ *p*-hydroxibenzoic acid acid: 0.11 mg·100 g^−1^ *p*-Coumaric acid: 0.07 mg·100 g^−1^ Naringenin: 0.31 mg·100 g^−1^ Vanillic acid: 0.07 mg·100 g^−1^ ꞵ-Carotene: 0.02 mg·100 g^−1^ ꞵ-Sitosterol≈ 1000 mg·100 g^−1^ Stigmasterol≈ 1000–1500 mg·100 g^−1^ Campesterol≈ 50 mg·100 g^−1^ α-tocopherol≈ 5 mg·100 g^−1^ γ-tocopherol≈ 2000 mg·100 g^−1^ δ-tocopherol≈ 5 mg·100 g^−1^	[74]
Passion seeds	284	56	1.02	n.d.	2.67	Isovitexin: 90.78 mg·g^−1^ Ferulic acid: 31.08 mg·g^−1^ *p*-coumaric: 24.74 mg·g^−1^ Gallic acid: 13.99 mg·g^−1^ Isorhmnetin: 7.40 mg·g^−1^ Quercetin: 3.05 mg·g^−1^	[75]

n.d.: not declared.

**Table 5:** Applications of UAE for extraction of fruit seed oils.

Food Waste	Solvent	Processing Conditions	Oil Recovery (wt%)	Bioactive Compounds	References
Cactus fruit seeds	*n*-hexane	Ultrasound intensity: 150–600 W Time: 60 min; Temperature: 40 °C Solid–solvent ratio 1:10 (*w*/*v*)	20.28 ± 0.68	α-tocopherol: 20.01–36.94 mg·100 g^−1^ γ-tocopherol: 12.73–19.15 mg·100 g^−1^ δ-tocopherol: 0.88–2.95 mg·100 g^−1^ Sinapic acid: 0.87–1.31 μg·g^−1^ Syringic acid: 0.14–0.15 μg·g^−1^ Salicylic acid: 0.21–0.22 μg·g^−1^ Palmitic acid: 10.01–12.71% Stearic acid: 5.14–6.33% Oleic acid: 10.04–11.46% Linoleic acid: 57.46–61.93% Linolenic acid: 5.41–7.20%	[45]
Passion seeds	ethanol	Power: 165 W; Frequency: 25 kHz Time: 10–50 min; Temperature: 30–60 °C Solid–solvent ratio 2–4 mL·g^−1^	12.32–21.76	Palmitic acid: 12.31% Palmitoleic acid: 0.19% Oleic acid: 17.03 Stearic acid: 2.44 Linoleic acid: 61.07 Linolenic acid: 0.12 Stigmasterol: 75.67 mg·100 g^−1^ Campesterol: 21.55 mg·100 g^−1^ ꞵ-Sitosterol: 69.11 mg·100 g^−1^	[11]
Apple seeds	*n*-hexane	Ultrasound power: 18–70 W Amplitude: 20–80%, Temperature: 30 °C Solid–solvent ratio 1:15 (*w*/*v*)	17.20 ± 2.3	Phloretin and phloridzin	[39]
Passion seeds	supercritical CO_2_	Ultrasound power: 160 W Pressure: 16 MPa, Temperature: 40 (*w*/*v*)	29	γ -tocopherol: 6.7–10.6 mg·100 g^−1^ γ-tocotrienol: 24–36 mg·100 g^−1^ δ-tocotrienol: 33–45 mg·100 g^−1^ Palmitic acid: 10.9–11.8% Oleic acid: 15.89–19% Linoleic acid: 63–67.91%	[12]
Mango seeds	*n*-hexane	Power: 70 W; Frequency: 42 kHz Time: 20–60 min; Temperature: 35–55 (*w*/*v*) Solid–solvent ratio 1:5 (*w*/*v*)	96.6 ± 1.3	Palmitic acid: 4.95% Stearic acid: 39.59% Oleic acid: 32.38% Linoleic acid: 3.31% Eicosadienoic acid: 2.32% Docosahexaenoic acid: 15.74%	[13]
Pomegranate seeds	water	Power: 130 W; Frequency: 20 kHz Temperature: 35–55 °C Time: 2–8 h	4.29–25.11	n.d.	[27]
Citrus seeds	*n*-hexane	Temperature: 25 °C Time: 90 min	Lime seeds: 22.07–22.08 Sweet orange seeds: 22.84–23.11	Lime seed TPC: ≈ 0.6–0.8 mg GAE·g^−1^ Palmitic acid: 19.83–20.79% Oleic acid: 17.27–18.07% Linoleic acid: 34.07–34.25% α-Linolenic acid: 10.96–11.45% Orange sweet seed TPC: ≈0.6–1 mg GAE·g^−1^ Palmitic acid: 23.56–23.73% Oleic acid: 20.95–21.58% Linoleic acid: 35.74–36.32% α-Linolenic acid: 3.67–3.98%	[30]
Bitter apricot kernels	natural deep eutectic solvent (choline chloride–lactic acid)	Liquid-to-solid ratio: 18 mL·g^−1^; Ultrasonic power: 420 W; Temperature: 42 °C; Extraction time: 32 min	45.64 ± 0.24	Palmitic acid:16.63 ±0.52 mg·g^−1^ Palmitoleic acid: 3.15 ± 0.16 mg·g^−1^ Ginkgolic acid: 0.62 ± 0.02 mg·g^−1^ Stearic acid: 3.86 ± 0.20 mg·g^−1^ Oleic acid: 309.18 ± 9.47 mg·g^−1^ Linoleic acid: 109.40 ± 8.35 mg·g^−1^ Arachidic acid: 0.36 ± 0.01 mg·g^−1^ *cis*-11-eicosenoic acid: 0.42 ± 0.02 mg·g^−1^ Octadecatrienoic acid: 0.60 ± 0.01 mg·g^−1^	[78]
Grape seeds	natural deep eutectic solvent (menthol–thymol)	Sample-to-solvent ratio: 1:4 Ultrasound sonicator (20 kHz, 400 W, and 70% amplitude) Temperature: 70 °C Time extraction: 20 min		Palmitic acid: 14.47% Stearic acid: 5.48% Oleic acid: 15.49% Linoleic acid: 56.17% α-linolenic acid: 5.18% TPC: 18.65 mg GAE·g^−1^ TFC: 0.73 mg RE·g^−1^	[79]

n.d.: not declared.

**Table 7:** Overview of the literature data on the application of PLE for obtaining fruit seed oils.

Extraction Method	Food Waste Material	Processing Conditions	Oil Recovery (wt%)	Content of Principal Bioactive Compounds	References
PLE	Avocado seeds	200 °C 11 MPa ethanol 20 min 5 g	39	Total polyphenols: 88 mg GAE·100 g^−1^	[48]
PLE	Avocado seeds	200 °C 11 MPa Ethanol–water 50:50 (*v*/*v*)	n.d	Organic acids, phenolic acids, flavonoids, catechins, and condensed tannins	[81]
Hot PLE	Grape pomace seeds	160 °C 10 atm 60% ethanol	n.d	Gallic acid: 21.66 μg·g^−1^ Ellagic acid: 4.55 μg·g^−1^ Protocatechuic acid: 5.31 μg·g^−1^ Caffeic acid: 4.92 μg·g^−1^ Chlorogenic acid: 6.66 μg·g^−1^ *p*-oumaric acid: 2.24 μg·g^−1^ Catechin: 85.91 μg·g^−1^ Epicatechin: 28.53 μg·g^−1^ Epigallocatechin: 13.21 μg·g^−1^ Gallocatechin: 3.27 μg·g^−1^	[87]
PLE	Berry fruit seeds	150 °C 10.34 MPa hexane 4 cycles × 5 min	16.00 (cranberry) 13.53 (strawberry) 5.08 (chokeberry) 7.60 (black currant) 1.70 (red currant)	Palmitic acid: 5.54–9.14% Stearic acid: 1.23–2.62% Palmitoleic acid: 0.11–0.36% Elaidic acid: 13.73–23.52% Linoleic acid: 36.35–67.23% γ-Linoleic acid: 0.12–8.72% Linolenic acid: 1.99–31.80%	[88]

n.d.: not declared.

**Table 8:** Overview of the hybrid techniques applied for obtaining fruit seed oils.

Extraction Method	Food Waste Material	Processing Conditions	Oil Recovery	Content of Principal Bioactive Compounds	References
SFE-UAE	Passion seeds	Temperature: 40–50 °C Pressure: 16–29 MPa CO_2_ flow rate: 0.63 kg·h^−1^ Solvent-seed mass ratio: 210 kg CO_2_·g^−1^ Ultrasound power: 160–800 W Time: 20 min	13.5–20.9%	γ-tocopherol: 6.7–9.4 mg·100 g^−1^ γ-tocotrienol: 24–34 mg·100 g^−1^ δ-tocotrienol: 33–44 mg·100 g^−1^ Palmitic acid: 10.9–11.6% Stearic acid: 3.01–5% Oleic acid: 15.89–19% Linoleic acid: 63–68.2%	[12]
PEF-SFE	Grape seeds	Solvent-seed mass ratio: 1/2 Temperature: 35–45 °C Pressure: 35–50 MPa CO_2_ flow rate: 0.9–2.7 kg·h^−1^	70–80 g·kg^−1^	Campesterol: 370–395.6 mg·kg^−1^ Stigmasterol: 508.8–527.2 mg·kg^−1^ β-Sitosterol: 3784.4–3973.9 mg·kg^−1^ α-tocopherol: 63.1–73.6 mg·kg^−1^ α-tocotrienol: 60.9–77.8 mg·kg^−1^ γ-tocopherol: 16.1–20.5 mg·kg^−1^ γ-tocotrienol: 88.2–108.1 mg·kg^−1^ Oleic acid: 210.8–211.1 g·kg^−1^ Linoleic acid: 645.2–646.1 g·kg^−1^ Procyanidin dimer B1: 912.1–1338.0 µg·kg^−1^ (+)-Catequin: 293.8–450.3 µg·kg^−1^ Procyanidin dimer B1: 912.1–1338.0 µg·kg^−1^ p-coumaric acid: 130.6–282.0 µg·kg^−1^ Gallic acid: 130.0–582.5 µg·kg^−1^ Dihydroxibenzoic acid: 191.5–267.4 µg·kg^−1^	[9]
Enzyme-assisted pressing	Apricot seeds, flaxseeds, and grape seeds	Types of enzymes: pectolytic, cellulotic, and hemicellulotic; time and temperature during incubation: 60 °C and 3 h; pH of enzyme solution: 6	11.05–30.85%	Total carotenoids: 0.037–0.731 mg·kg^−1^ Total phenolic compounds: 31.08–127.92 mg·kg^−1^ α-tocopherol: 111.60–156.59 mg·L^−1^ β-tocopherol: 0.147–154.29 mg·L^−1^ γ-tocopherol: 413.00–619.04 mg·L^−1^ δ-tocopherol: 0.147–150.97 mg·L^−1^ Oleic acid: 18.10–69.28% Linoleic acid: 14.32–68.33% α-linoleic acid: 0.08–57.49%	[98]
UMAE	Mango seeds	Bath of ultrasonicator (42 kHz, 70 WUltrasound); Ultrasound extraction time: 20–60 min Ultrasound extraction temperature: 35–55 °C Type of solvent: *n*-hexane Mass–solvent ratio: 1:3–1:8 Power MAE: 250–350 W MAE time: 5–15 min	11.60%	Stearic acid: 39.59% Oleic acid: 32.33% Docosahexaenoic acid: 15.74%	[13]
HCUEPM	*Litsea cubeba* seeds	Types of enzymes: hemicellulose, pectinase, and neutral protease Incubation temperature: 30–50 °C	204.23 mL·kg^−1^	*D*-Limonene: ND-0.48% Lauric acid: 48.72–57.47% Decanoic acid: 10.89–11.67% Oleic acid: 8.93–14.37% Linoleic acid: 4.28–7.69%	[50]
UMAEE	Cherry seeds	Ultrasonic power: 560 W, Microwave power: 323 W for 38 min at 40 °C Enzyme: 2.7% (cellulase, hemicellulase, and pectinase)	83.85%	Palmitic acid: 5.68% Oleic acid: 53.82% Linoleic acid: 38.29%	[99]

**Table 9:** AGREEprep theoretical simulation of fruit oil extraction techniques.

Extraction Techniques	Food Waste Material	Analytes	Processing Conditions	Analytical Techniques	Criterion	Metrics	References
UAE	Passion seeds	Sterols and fatty acids	Power 165 W, frequency 25 kHz, time 10–50 min, temperature 30–60 °C, and sample–solvent ratio 2–4 mL·g^−1^	GC-MS	Criterion 1: ex situ Criterion 2: non-hazardous materials Criterion 3: only sustainable materials Criterion 4: no waste Criterion 5: 1 g of sample Criterion 6: 4 sample throughput Criterion 7: 2 steps or fewer and semi-automated Criterion 8: 82.5 W·h^−1^ Criterion 9: liquid chromatography; gas chromatography with quadrupole detection Criterion 10: non-hazardous or no exposure	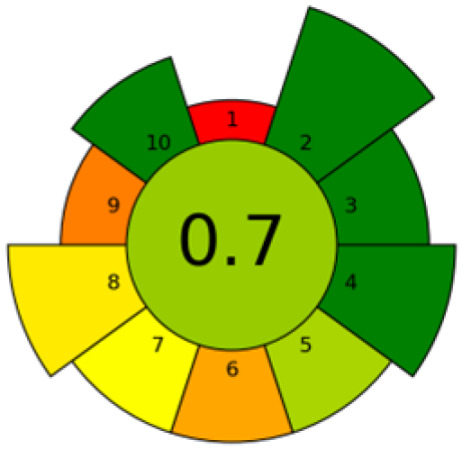	[11]
MAE	Pomegranate seeds	Fatty acids, sterols, and tocopherols	Sample–solvent ratio: 1:6 (*w*/*v*) 500 W, and frequency of 40 kHz for 9 min	GC-FID HPLC-FLD	Criterion 1: ex situ Criterion 2: non-hazardous materials Criterion 3: only sustainable materials Criterion 4: no waste Criterion 5: 1 g of sample Criterion 6: 10 sample throughput Criterion 7: 3 steps or fewer and semi-automated Criterion 8: 41.65 W·h^−1^ Criterion 9: liquid chromatography; gas chromatography with quadrupole detection Criterion 10: 1 hazard	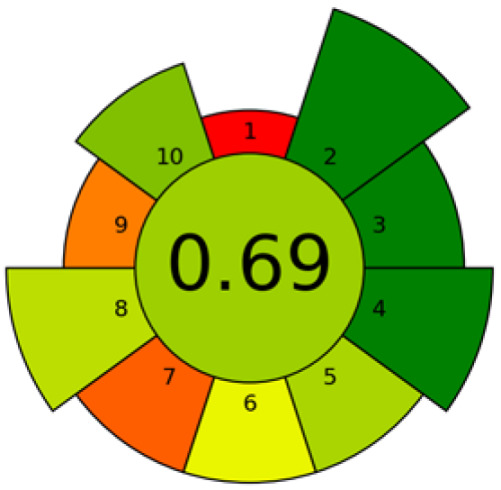	[26]
EAE	Mango seeds	Fatty acid	15 g of seeds Sample–water ratio: 1:10 (*w*/*v*) 5 min 2% of enzymes	GC-FID	Criterion 1: ex situ Criterion 2: non-hazardous materials Criterion 3: only sustainable materials Criterion 4: no waste Criterion 5: 15 g of sample Criterion 6: 12 sample throughput Criterion 7: 2 steps or fewer and manual systems Criterion 8: 69.4 W·h^−1^ Criterion 9: gas chromatography with non-MS detection Criterion 10: non-hazardous or no exposure	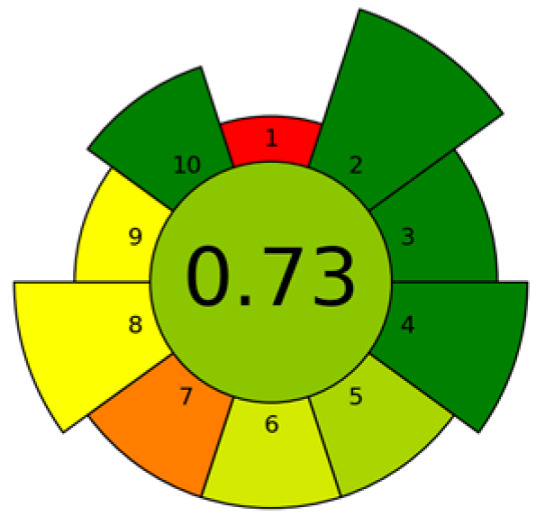	[107]
PLE	Radish seeds	Phytosterols and tocopherols	5.2 g of seeds 120, 135, or 150 °C 5, 10, or 15 MPa 10 or 30 min	GC-MS	Criterion 1: ex situ Criterion 2: non-hazardous materials Criterion 3: only sustainable materials Criterion 4: no waste Criterion 5: 15 g of sample Criterion 6: 12 samples throughout Criterion 7: 2 steps or fewer and manual systems Criterion 8: 250 W·h^−1^ Criterion 9: liquid chromatography; gas chromatography with quadrupole detection Criterion 10: non-hazardous or no exposure	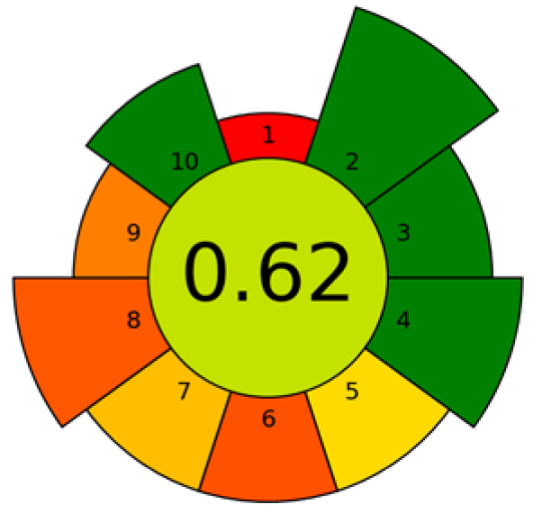	[108]
PEF	Berry seeds	Fatty acids	2 g of seeds 8 or 10 kV electrode voltage, 7 µs pulse width, 20 Hz frequency, and an adequate number of pulses to achieve energy intake of 50 kV kg^−1^ 30 min	GC-FID	Criterion 1: ex situ Criterion 2: non-hazardous materials Criterion 3: only sustainable materials Criterion 4: no waste Criterion 5: 2 g of sample Criterion 6: 2 sample throughput Criterion 7: 2 steps or fewer and semi-automated Criterion 8: 13.89 W·h^−1^ Criterion 9: gas chromatography with non-MS detection Criterion 10: non-hazardous or no exposure	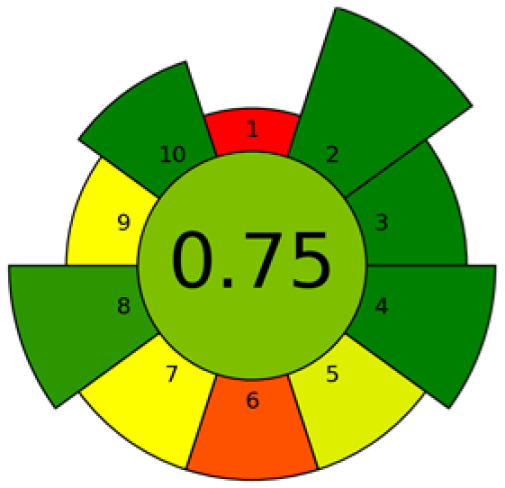	[93]
Cold pressing	Cherry seeds	Fatty acids and tocopherols	6000 g of seed 70 °C	GC-MS and HPLC-FLD	Criterion 1: ex situ Criterion 2: non-hazardous materials Criterion 3: only sustainable materials Criterion 4: no waste Criterion 5: 2000 g of sample Criterion 6: 6 sample throughput Criterion 7: 2 steps or fewer and semi-automated Criterion 8: 200 W·h^−1^ Criterion 9: gas chromatography with non-MS detection Criterion 10: non-hazardous or no exposure	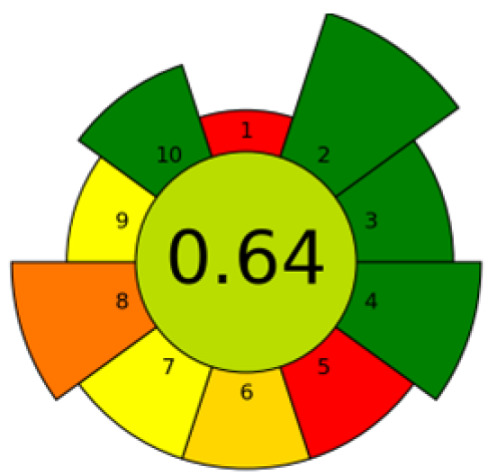	[40]
Soxhlet extraction	Passion seeds	Fatty acids and tocopherols	10 g of seed Sample–solvent ratio: 1:20 (*w*/*v*) 4 h 60 °C	GC-MS and HPLC-DAD	Criterion 1: ex situ Criterion 2: 200 mL of hazardous materials Criterion 3: >75% sustainable materials Criterion 4: 200 mL of waste Criterion 5: 10 g of sample Criterion 6: 0.25 sample throughput Criterion 7: 3 steps or fewer and semi-automated Criterion 8: 2000 W·h^−1^ Criterion 9: liquid chromatography; gas chromatography with quadrupole detection Criterion 10: 1 hazard	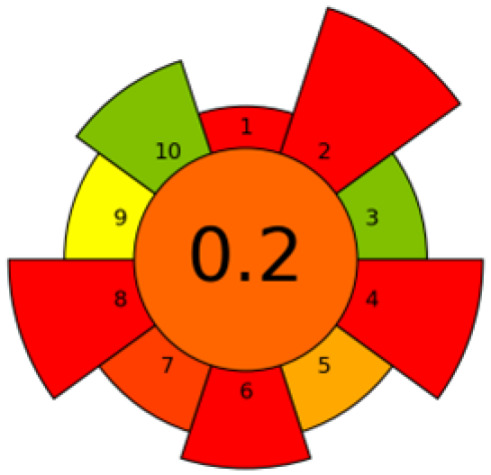	[24]
Supercritical CO_2_ extraction	Berry seeds	Fatty acids and tocopherols	14 kg of seed sample 1000 bar 100 °C 240 kg·h^−1^	RP-HPLC-FLD	Criterion 1: ex situ Criterion 2: non-hazardous materials Criterion 3: only sustainable materials Criterion 4: no waste Criterion 5: 14,000 g of sample Criterion 6: 1 sample throughput Criterion 7: 3 steps and semi-automated Criterion 8: 3000 W·h^−1^ Criterion 9: liquid chromatography; gas chromatography with quadrupole detection Criterion 10: no hazards	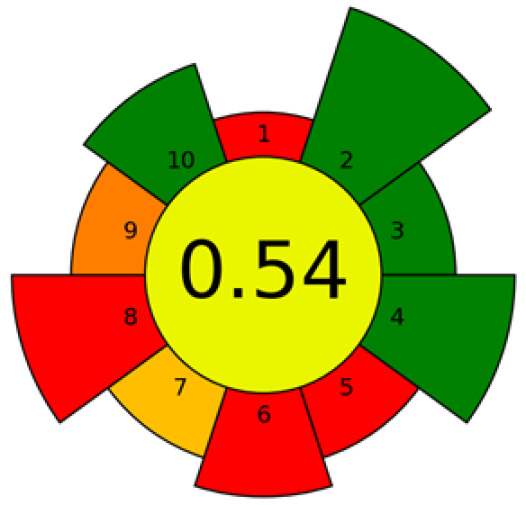	[73]

## Data Availability

No new data were created or analyzed in this study. Data sharing is not applicable to this article.

## Abbreviations

The following abbreviations are used in this manuscript:

AGREE	Analytical Greenness Metric Approach
AGREEprep	Analytical Greenness Metric for Sample Preparation
AI	Artificial intelligence
ANN	Artificial neural network
BAGI	Blue Applicability Grade Index
CLCAs	Life Cycle Assessments
ACLCAs	Attributional Life Cycle Assessments
DAD	Diode-array detector
EAE	Enzyme-assisted extraction
EFSA	European Food Safety Authority
FDA	Food and Drug Administration
FAMEs	Fatty acid methyl esters
FID	Flame ionization detection
GAC	Green Analytical Chemistry
GC	Gas chromatography
GAMs	Green Analytical Metrics
GAPI	Green Analytical Procedure Index
HCUEPM	Homogenization-circulating ultrasound with enzymatic pretreatment microwave-assisted extraction
HPLC	High-performance liquid chromatography
MAE	Microwave-assisted extraction
MAEB	Microwave-assisted extraction based on emulsion breaking
MS	Mass spectrometry
MUFAs	Monounsaturated fatty acids
NEMI	National Index of Environmental Methods
PEFs	Pulsed electric fields
PEF-SFE	Pulsed electric field–supercritical fluid extraction
PLE	Pressurized liquid extraction
PUFAs	Polyunsaturated fatty acids
RSM	Response surface methodology
ScCO_2_	Supercritical carbon dioxide extraction
SDGs	Sustainable Development Goals
SFAs	Saturated fatty acids
S/F	Solvent-to-sample feed mass ratio
SFE	Supercritical fluid extraction
SFE-UAE	Supercritical fluid extraction–ultrasound-assisted extraction
TEA	Technical–economic analysis
TPC	Total polyphenol content
TFC	Total flavonoid content
UAE	Ultrasound-assisted extraction
UFAs	Unsaturated fatty acids
UMAE	Ultrasound–microwave assisted extraction
UMAEE	Ultrasound–microwave-assisted aqueous enzymatic extraction
